# The AhR-Ovol1-Id1 regulatory axis in keratinocytes promotes epidermal and immune homeostasis in atopic dermatitis-like skin inflammation

**DOI:** 10.1038/s41423-025-01264-z

**Published:** 2025-02-13

**Authors:** Zeyu Chen, Morgan Dragan, Peng Sun, Daniel Haensel, Remy Vu, Lian Cui, Peiyao Zhu, Nan Yang, Yuling Shi, Xing Dai

**Affiliations:** 1 Department of Dermatology, Shanghai Skin Disease Hospital, Tongji University School of Medicine, Shanghai, China; 2 Department of Biological Chemistry, School of Medicine, University of California, Irvine, CA 92697 USA; 3 Department of Dermatology, Shanghai Tenth People’s Hospital, Tongji University School of Medicine, Shanghai, China; 4 Institute of Psoriasis, Tongji University School of Medicine, Shanghai, China; 5 The NSF-Simons Center for Multiscale Cell Fate Research, University of California, Irvine, CA 92697 USA; 6 Department of Dermatology, School of Medicine, University of California, Irvine, CA 92697 USA

**Keywords:** AhR, Ovol1, Id1, Transcription factor, Atopic dermatitis, Epidermis, Skin inflammation, Skin barrier, IL-1, Gamma delta T, Innate immunity, Mechanisms of disease

## Abstract

The skin is our outer permeability and immune defense barrier against myriad external assaults. Aryl hydrocarbon receptor (AhR) senses environmental factors and regulates barrier robustness and immune homeostasis. AhR agonists have been approved by the FDA for psoriasis treatment and are in clinical trials for the treatment of atopic dermatitis (AD), but the underlying mechanism of action remains poorly defined. Here, we report that *OVOL1/Ovol1* is a conserved and direct transcriptional target of AhR in epidermal keratinocytes. We show that OVOL1/Ovol1 influences AhR-mediated regulation of keratinocyte gene expression and that *OVOL1/Ovol1* ablation in keratinocytes impairs the barrier-promoting function of AhR, exacerbating AD-like inflammation. Mechanistically, we have identified Ovol1’s direct downstream targets genome-wide and provided in vivo evidence supporting the role of *Id1* as a functional target in barrier maintenance, disease suppression, and neutrophil accumulation. Furthermore, our findings reveal that an IL-1/dermal γδT cell axis exacerbates type 2 and 3 immune responses downstream of barrier perturbation in *Ovol1*-deficient AD skin. Finally, we present data suggesting the clinical relevance of OVOL1 and ID1 functions in human AD skin. Our study highlights a keratinocyte-intrinsic AhR-Ovol1-Id1 regulatory axis that promotes both epidermal and immune homeostasis in the context of skin inflammation, identifying new therapeutic targets.

## Introduction

Atopic dermatitis (AD) is the most common inflammatory skin disease and affects 15–20% of children and up to 10% of adults [1]. Environmental allergens such as house dust mites (HDMs) are common triggers, whereas a defective skin epidermal barrier caused by genetic mutations (e.g., *FLG*) and/or inflammatory cytokines is a salient feature, of AD [2–7]. AD skin is usually heavily colonized by the pathogenic bacteria *Staphylococcus aureus*, which further aggravates the epidermal barrier and immune defects to perpetuate disease progression [2]. Type 2 immunity, characterized by the production of T helper 2 (Th2)-related cytokines (e.g., IL-4 and IL-13) and the activation of eosinophil/mast cells [8], contributes to AD pathogenesis by impairing the epidermal barrier and causing symptoms such as inflammation and itch [5–7, 9–11]. Emerging evidence suggests that type 3 immunity, characterized by the production of Th17-related cytokines (e.g., IL-17A) and the recruitment of neutrophils, is also involved in AD development, especially in intrinsic, pediatric or Asian patients, by fueling the type 2 immune response [3, 8, 12, 13]. Biologics that target IL-4/IL-13 or small molecule inhibitors that target JAK/STAT signaling have shown efficacy in treating AD patients [14, 15]. However, not all patients respond optimally to these treatments. Further advancements in therapy require a deeper understanding of the molecular and cellular mechanisms underlying AD-associated barrier dysregulation and inflammation. In particular, epidermal-intrinsic mechanisms that increase barrier strength while regulating early immunological responses to AD-associated allergen/pathogen attacks are ideal therapeutic targets given the potential for topical treatment, but such mechanisms remain largely elusive.

Aryl hydrocarbon receptor (AhR), an environment-sensing xenobiotic receptor and ligand-activated transcription factor vital for skin immune homeostasis and barrier maintenance, has attracted attention as a therapeutic target for both AD and psoriasis [16–20]. Specifically, the AhR agonist tapinarof has recently been approved by the FDA as a first-of-its-kind topical drug for psoriasis and is in clinical trials for AD. This shared efficacy implies that AhR is involved in early and common events of skin inflammation. However, surprisingly little is known regarding the cellular and molecular targets of AhR, the activation of which can elicit both anti-inflammatory and proinflammatory consequences in animal and patient studies [21–25]. Thus, dissecting the downstream mediators, especially the anti-inflammatory arm, of the AhR function in the skin is important from both basic science and clinical perspectives.

In this study, we identified *OVOL1*/*Ovol1* as a direct transcriptional target of AhR that, in turn, modifies AhR regulation of epidermal keratinocyte gene expression and barrier function. Human *OVOL1* encodes a zinc finger transcriptional repressor and has been identified by genome-wide association studies to be an AD and acne risk locus [26–28]. Mouse *Ovol1* promotes epidermal cell cycle arrest and suppresses psoriasis-like skin inflammation [29–32]. Here, we show that keratinocyte-specific deficiency of *Ovol1* exacerbates AD-like inflammation induced by treatment with HDM and *Staphylococcus aureus*-derived toxin staphylococcal enterotoxin B (SEB), two environmental agents relevant to human AD pathogenesis [2] that are known to induce AD-like phenotypes in mice when used in combination [33]. We found that Ovol1 does so by repressing the expression of myriad downstream targets, including the transcription factor-encoding *Id1*, the inhibition of which partially rescues *Ovol1* deficiency-associated barrier disruption and neutrophil infiltration. We also identified dermal gamma delta T (γδT) cells and IL-1 signaling as critical cellular and molecular mediators of AD-like inflammation downstream of *Ovol1* deficiency-induced barrier dysregulation. Finally, we present data suggesting the clinical importance of OVOL1 and ID1 functions in human AD. Our study highlights a keratinocyte-intrinsic AhR-Ovol1-Id1 regulatory axis that functions at the environment-barrier-immune interfaces to protect skin homeostasis against skin inflammation and implicates new therapeutic targets for both AD and psoriasis.

## Results

### AhR directly activates *OVOL1* expression in human keratinocytes to suppress the cell cycle and promote differentiation

**Fig. 1:**
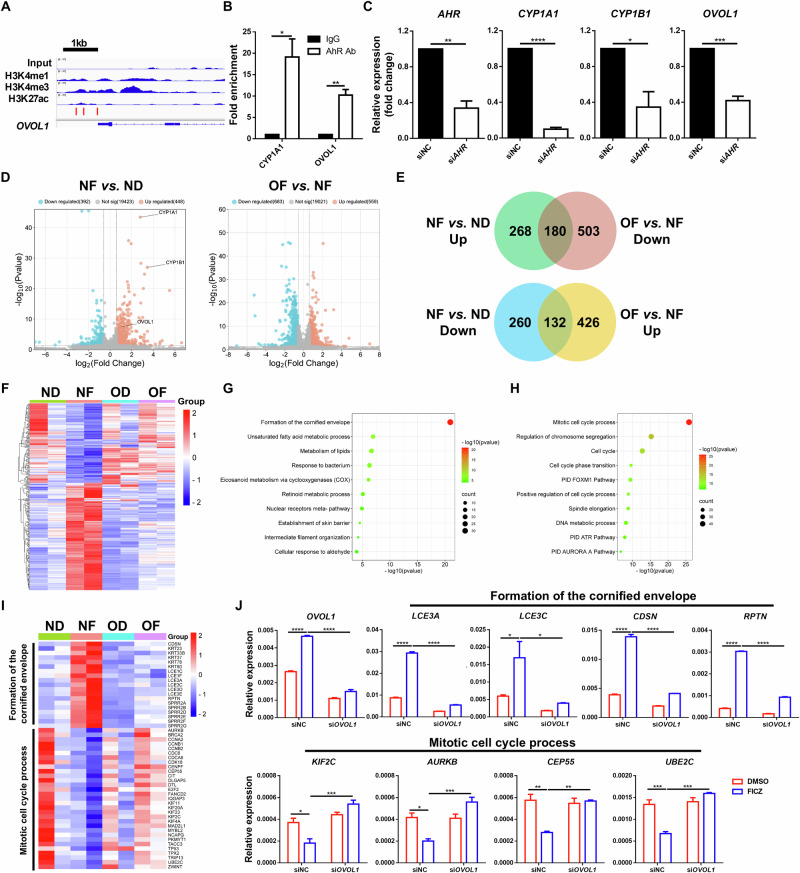
*OVOL1* is a direct target of AhR and affects its regulation of gene expression in human keratinocytes. **A** Genome browser track for the indicated ChIP-seq signals across the *OVOL1* locus. The red bar indicates the presence of the AhR binding motif (GCGTG). **B** ChIP‒qPCR results of the indicated genes in FICZ-treated NHEKs. The IgG control values were normalized to 1. **C** RT‒qPCR results of the indicated genes in scrambled or *AHR* siRNA-treated NHEKs. **D** Volcano plots showing differential gene expression in the indicated samples. ND: siNC (negative control) + DMSO; NF: siNC + FICZ; OD: si*OVOL1* + DMSO; OF: si*OVOL1* + FICZ. **E** Venn diagrams of differentially expressed genes (DEGs) showing overlap between FICZ-induced genes (NF *vs*. ND) and OVOL1-dependent genes (OF *vs*. NF). UP upregulated. Down, downregulated. The numbers of DEGs are indicated. **F** Heatmap of the DEGs from **E**. **G**, **H** Ingenuity pathway analysis of the 180 (**G**) or 132 (**H**) overlapping DEGs from **E**. **I** Heatmap of DEGs in the indicated pathways. **J** RT‒qPCR results of the indicated genes in DMSO- or FICZ-treated NHEKs with or without *OVOL1* knockdown. The results are summarized from 3 (**B**, **C**, **J**) or 2 (**D**) independent experiments. For **B**, **C** and **J**, the data are presented as the means ± SEMs. *p < 0.05, **p < 0.01, ***p < 0.001, ****p < 0.0001. *p* values were calculated via 2-tailed unpaired Student’s *t* test (**B** and **C**) or two-way ANOVA (**J**)

Chemical activation of AhR in cultured normal human epidermal keratinocytes (NHEKs) upregulates *OVOL1* [26], but the underlying regulatory mechanism is unknown. We examined the sequence of the *OVOL1* promoter and identified several AhR-binding motifs (GCGTG) (Fig. 1A). ChIP‒qPCR analysis of NHEKs revealed that AhR physically binds to these sites, as it does to *CYP1A1*, a well-known AhR downstream target [34] (Fig. 1B). Furthermore, small interfering RNA (siRNA)-mediated silencing of *AHR* expression in NHEKs resulted in significant downregulation of not only the *AHR* canonical targets *CYP1A1* and *CYP1B1* [34] but also *OVOL1* mRNA expression (Fig. 1C). These results demonstrate that *OVOL1* is a direct transcriptional target of the endogenous AhR protein in human keratinocytes.

Next, we assessed the importance of OVOL1 in mediating AhR function in human keratinocytes. Treatment of NHEKs with the AhR agonist 6-formylindolo[3,2-b]carbazole (FICZ) led to increased and decreased expression of 448 and 392 genes, respectively, in the presence of a scrambled siRNA, with *OVOL1*, *CYP1A1*, and *CYP1B1* being among the elevated genes (Fig. 1D, Supplementary Fig. S1A, Supplementary Table S1). Metascape analysis of the FICZ-induced genes revealed “skin development”, “skin epidermis development”, and “metabolism of lipids” as the top terms (Supplementary Fig. S1B, Supplementary Table S2), whereas the FICZ-suppressed genes were enriched for “mitotic cell cycle process”, “cell cycle”, and “positive regulation of cell migration” terms (Supplementary Fig. S1C, Supplementary Table S3). The knockdown of *OVOL1* in DMSO-treated NHEKs increased the expression of 304 genes and decreased the expression of 542 genes (Supplementary Fig. S1D, E, Supplementary Table S4). The downregulated genes were enriched for “skin development”, “positive regulation of hydrolase activity”, and “metabolism of lipids” (Supplementary Fig. S1F, Supplementary Table S5), whereas the upregulated genes were enriched for “cytokine signaling in the immune system”, “network map of the SARS-CoV-2 signaling pathway”, and “rhythmic process” (Supplementary Fig. S1G, Supplementary Table S6). These results reveal both overlapping (skin development and lipid metabolism) and divergent (cell cycle control *vs*. immune modulation) functions of AhR and OVOL1 in human keratinocytes.

We then examined how FICZ shapes OVOL1 function and vice versa. We found that *OVOL1* knockdown in FICZ-treated NHEKs increased the expression of 559 genes and decreased the expression of 683 genes (Fig. 1D, Supplementary Fig. S1H; Supplementary Table S7). The downregulated genes were enriched for “keratinization”, “NOD-like receptor signaling pathway”, and “IL-18 signaling pathway” (Supplementary Fig. S1I, Supplementary Table S8). The upregulated genes were enriched for “mitotic cell cycle”, “cell cycle”, and “positive regulation of cell cycle process” (Supplementary Fig. S1J, Supplementary Table S9). These results, together with the findings above, suggest a partial shift in the molecular function of OVOL1, from predominantly regulating immune activity under homeostatic conditions to suppressing the cell cycle when AhR signaling is activated by an exogenous ligand. Among the 448 genes upregulated and 392 genes downregulated by FICZ alone, 40.2% (180/448) and 33.7% (132/392), respectively, were dependent on *OVOL1* expression (Fig. 1E, F, Supplementary Table S10), underscoring the critical importance of *OVOL1* in AhR-directed gene expression. Interestingly, FICZ induction of *CYP1A1*, but not *CYP1B1*, was dependent on *OVOL1* (Supplementary Table S10). The top enriched pathways in the FICZ-induced, *OVOL1*-dependent genes were “formation of the cornified envelope”, “unsaturated fatty acid metabolic process”, and “metabolism of lipids” (Fig. 1G, I; Supplementary Table S11). The top enriched terms in the FICZ-suppressed, *OVOL1*-dependent genes were “mitotic cell cycle process”, “regulation of chromosome segregation”, and “cell cycle” (Fig. 1H, I, Supplementary Table S12). RT‒qPCR analysis confirmed the failure of FICZ to upregulate genes involved in the “formation of the cornified envelope” pathway (e.g., *LCE3A*, *LCE3C*, *CDSN* and *RPTN*) and downregulated genes involved in the “mitotic cell cycle process” pathway (e.g., *KIF2C*, *AURKB*, *CEP55* and *UBE2C*) after *OVOL1* depletion (Fig. 1I, J). These data show that activated AhR signaling in human keratinocytes requires *OVOL1* to promote the expression of genes associated with epidermal differentiation and lipid metabolism and to inhibit the expression of genes associated with the cell cycle.

To further investigate the correlation between AhR and OVOL1 function in human keratinocytes, we silenced *AHR* in NHEKs to compare it with *OVOL1* deficiency-induced molecular changes. The knockdown of *AHR* resulted in the downregulation of 233 genes and the upregulation of 380 genes (Supplementary Table S13), with 17.2% (40/233) of the downregulated genes and 25.8% (98/380) of the upregulated genes overlapping with genes regulated by OVOL1 (Supplementary Fig. S1K, L, Supplementary Tables S14, 15). The common downregulated genes were enriched for “cholesterol biosynthesis”, “keratinization”, and “cellular response to metal ions” (Supplementary Fig. S1M, Supplementary Table S16). The commonly upregulated genes were enriched for “interferon alpha/beta signaling”, “cellular response to mineralocorticoid stimulus”, and “regulation of nervous system development” (Supplementary Fig. S1N, Supplementary Table S17). These data support the idea that both AhR and OVOL1 regulate processes such as lipid metabolism, differentiation, and immune-associated responses in human keratinocytes.

Collectively, our findings suggest a mechanistic model in which AhR directly activates the expression of *OVOL1*, which in turn affects the ability of AhR to reprogram keratinocyte gene expression from a pro-proliferation state to a pro-differentiation state.

### AhR activates *Ovol1* expression in mouse keratinocytes and relies on *Ovol1* for its downstream in vivo function in promoting barrier robustness against AD stimuli

**Fig. 2:**
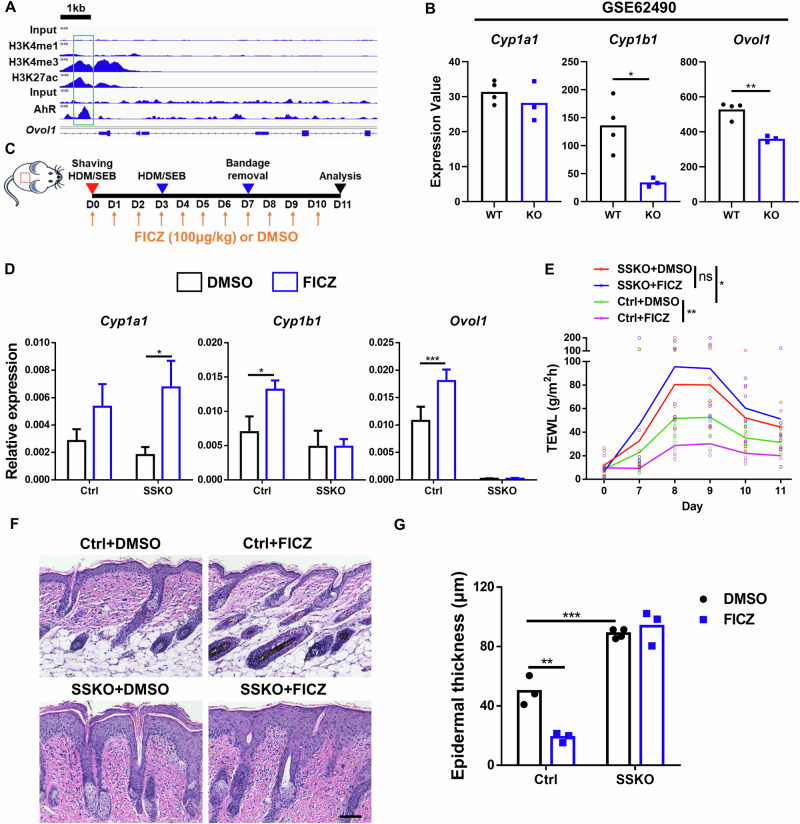
AhR requires *Ovol1* to mediate target activation and barrier maintenance in mice. **A** Genome browser tracks for the indicated ChIP-seq signals across the *Ovol1* locus. The green box highlights the AhR-bound region. **B** RNA-seq results of the indicated genes in mouse primary keratinocytes from *Ahr*^+/+^ (n = 4) or *Ahr*^−/−^ (n = 3) mice. **C** Experimental design for FICZ treatment in **D**–**G**. DMSO was used as a vehicle control. **D** RT‒qPCR results of the indicated genes in whole skin on day 11. Data are presented as the means ± SEMs. n = 4 mice per group. **E** Time course showing TEWL measurements. n = 5–9 mice per group. **F** Representative skin histology (H&E staining) on day 11. Scale bar = 100 μm. **G** Quantification of epidermal thickness. n = 3–4 mice per group. For **D**, the data are presented as the means ± SEMs. *p < 0.05, **p < 0.01, ***p < 0.001. ns nonsignificant. *p* values were calculated via 2-tailed unpaired Student’s *t* test (**B**) or two-way ANOVA (**D**, **E** and **G**)

The dramatic differences between human and mouse skin [35, 36] suggest that conservation of regulatory mechanisms cannot be assumed. To determine whether *Ovol1* is a direct target of AhR in mice, we analyzed published AhR ChIP-seq data from primary murine keratinocytes [37] and found that AhR directly binds to the mouse *Ovol1* promoter (Fig. 2A). Interestingly, interrogation of published RNA sequencing (RNA-seq) data on primary keratinocytes from *Ahr*^+/+^ and *Ahr*^−/−^ mice [38] revealed significantly decreased expression of *Cyp1b1* and *Ovol1* but not other known AhR targets, such as *Cyp1a1, Bax* [39]*, Cdkn1b* [40]*, Gsta1, Gsta2* [41] *and Hsp90aa1* [17], in *Ahr*^−/−^ keratinocytes (Fig. 2B, Supplementary Fig. S2A). Moreover, analysis of published RNA-seq data from the epidermis of newborn wild-type (WT) and constitutively active AhR (AhR-CA) mice [37] revealed a significant overlap in the genes upregulated by AhR-CA and FICZ, including *Ovol1* and other known AhR targets (Supplementary Fig. S2B). Therefore, the direct activation of *Cyp1a1*/*CYP1A1*, *Cyp1b1*/*CYP1B1*, and *Ovol1/OVOL1* by AhR in epidermal keratinocytes is likely evolutionarily conserved, although not all AhR targets exhibit this response.

AhR signaling in keratinocytes promotes barrier integrity against AD-associated perturbations [17, 18, 23, 42], whereas AhR expression is significantly decreased in the AD epidermis (Supplementary Fig. S2C, D) [43]. Therefore, we asked whether *Ovol1* is required for the barrier-promoting function of AhR. We administered FICZ or DMSO via intraperitoneal (i.p.) injection to skin epithelium-specific *Ovol1* knockout (SSKO: *K14*-Cre;*Ovol1*^f/-^) mice and control littermates that were treated with HDM/SEB (Fig. 2C). As expected, FICZ was able to rescue skin barrier dysfunction and epidermal hyperplasia in a manner dependent on AhR expression in keratinocytes (Supplementary Fig. S2E–H). Moreover, FICZ elevated *Cyp1a1, Cyp1b1*, and *Ovol1* expression in the skin of control mice (Fig. 2D, Supplementary Fig. S2I), further indicating that these genes are bona fide AhR targets. The FICZ-induced upregulation of *Cyp1b1*, but not other AhR targets, was abolished in the SSKO mice (Fig. 2D, Supplementary Fig. S2J). ChIP‒qPCR and ChIP‒seq (see below) experiments revealed strong Ovol1 binding to the *Cyp1b1* promoter (Supplementary Fig. S2K, L), suggesting feedforward regulation (Supplementary Fig. S2M). Importantly, FICZ partially normalized trans-epidermal water loss (TEWL) in HDM/SEB-treated control mice but failed to reverse exacerbated barrier dysfunction in their SSKO counterparts (Fig. 2E). Histological analysis of skin sections revealed that FICZ treatment significantly reduced epidermal thickness in HDM/SEB-treated control mice but not in SSKO mice (Fig. 2F, G). Together, these in vivo data provide evidence that the function of AhR in target activation and barrier promotion depends on the expression of Ovol1 in keratinocytes.

### *Ovol1* in keratinocytes protects skin from AD-like barrier dysregulation and pathology

**Fig. 3:**
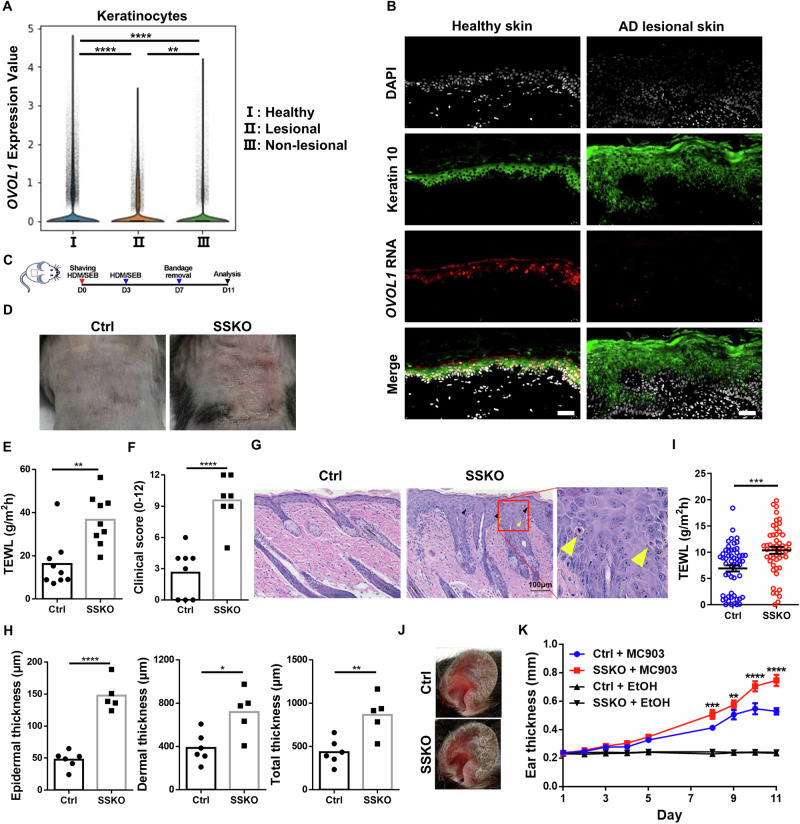
*Ovol1* deficiency in keratinocytes aggravates AD-like skin inflammation. **A** Violin plots of *OVOL1* expression in keratinocytes from healthy, lesional, and nonlesional skin. **B** Expression of the keratin 10 protein and *OVOL1* RNA in healthy and lesional skin (n = 10 per group). DAPI was used to stain the nuclei. Scale bar = 40 μm. **C** Experimental design for HDM/SEB treatment in **E**–**H**. **D** Representative photographs of HDM/SEB-treated control (Ctrl) and SSKO mice on day 11. **E** TEWL values of HDM/SEB-treated Ctrl (n = 9) and SSKO (n = 9) mice. **F** Clinical scores of HDM/SEB-treated Ctrl (n = 8) and SSKO (n = 7) mice. **G** Representative skin histology (H&E staining) on day 11. Scale bar = 100 μm. The black arrowheads indicate spongiosis. Yellow arrowheads indicate epidermal infiltration of eosinophils. **H** Quantification of epidermal, dermal and total skin thickness in Ctrl (n = 6) and SSKO (n = 5) mice. **I** TEWL values of untreated Ctrl (n = 55) and SSKO (n = 51) mice. **J** Representative photographs of MC903-treated Ctrl and SSKO mice on day 11. **K** Ear thickness (mean ± SEM) of MC903-treated Ctrl (n = 6) and SSKO (n = 6) mice. *p < 0.05, **p < 0.01, ***p < 0.001, ****p < 0.0001. *p* values were calculated via *t* tests followed by Benjamini‒Hochberg correc*t*ion (**A**), 2-tailed unpaired Student’s t tests (**E**, **F** and **H**), Mann‒Whitney U tests (**I**) or two-way ANOVA (**K**, Ctrl + MC903 *versus* SSKO + MC903)

Our discovery that *Ovol1* is a direct and functionally significant target of AhR raises the question of whether *Ovol1* itself plays a regulatory role in AD-like skin inflammation. To address this, we analyzed published single-cell RNA-seq data from whole-skin samples from healthy individuals and AD patients [44] and observed downregulated *OVOL1* expression in AD keratinocytes, especially in lesional skin (Fig. 3A, Supplementary Fig. S3A). In situ detection of *OVOL1* RNA validated its reduced expression in AD lesional skin epidermis (Fig. 3B). In support of the functional relevance of this observation, the deletion of *Ovol1* from mouse keratinocytes significantly aggravated the HDM/SEB-induced epidermal phenotypes, as evidenced by the more severe barrier disruption and epidermal hyperplasia in the SSKO mice than in their control littermates (Fig. 2E–G), findings that held true when the experiments were repeated with no DMSO (Fig. 3C–H). The TEWL values were greater and epidermal thickening is more prominent in HDM/SEB-treated SSKO mice than in control littermates, and the other AD-like skin phenotypes, as measured by the clinical scores of skin eruption, scaling, bleeding and redness as well as dermal thickening, were significantly more severe in SSKO mice than in their control littermates (Fig. 3C–H, Supplementary Fig. S3B).

At the histological level, the lesional skin of the SSKO mice, but not their control littermates, presented AD-like pathology, including spongiosis and epidermal infiltration of eosinophils (Fig. 3G, Supplementary Fig. S3C). Flow cytometry analysis revealed a trend toward an increase in the total number of Th2 cells, which play critical roles in AD pathogenesis [1, 3], per lymph node in SSKO mice, whereas the percentage of Th2 cells among CD4^+^ T cells was not significantly altered (Supplementary Fig. S3D, E). Compared with control skin, SSKO skin also contained more GATA3^+^ cells [GATA3 is a key transcription factor for Th2 cells [45]], particularly in regions enriched with T cells (CD3^+^) (Supplementary Fig. S3F). Interestingly, the protective role of Ovol1 against AD-like skin phenotypes was evident even after prolonged (3 rounds) exposure to HDMs and SEB (Supplementary Fig. S3G–K), suggesting a persistent long-term effect.

We also examined the impact of *Ovol1* deletion during homeostasis and using another AD model, namely, ear skin responses to MC903 [46, 47]. During homeostasis, a slight but statistically significant increase in TEWL was observed in the SSKO mice, despite the absence of overt skin phenotypes and histological alterations (Fig. 3I, Supplementary Fig. S3L). Consistently, molecular differences were detected between the epidermis of *Ovol1*^−/−^ and control littermates [30, 31] (Supplementary Fig. S3M–O, Supplementary Table S18–20). Compared with that of the control littermates, the skin of the MC903-treated SSKO mice was more symptomatic and significantly thicker (Fig. 3J, K). Compared with those of control littermates, the ears of *Ovol1*^−/−^ mice also presented worsened skin inflammation and were significantly thicker (Supplementary Fig. S3P, Q). When a high dose of MC903 was used on the right ear, even the left ear skin (sham control) and back skin of *Ovol1*^−/−^ mice were thicker and more proliferative than those of their control littermates were (Supplementary Fig. S3R–T), suggesting that local MC903 administration elicited a systemic effect in these mice. Interestingly, the serum levels of Cxcl2 and G-CSF, factors involved in neutrophil recruitment or maturation, were significantly elevated in *Ovol1*^−/−^ mice compared with their control littermates (Supplementary Fig. S3U). Moreover, neutrophils were observed in the skin of both the directly applied (right) and sham control (left) ears of MC903-treated *Ovol1*^−/−^ mice (Supplementary Fig. S3V, W).

Taken together, our data demonstrate the functional importance of *Ovol1* in suppressing AD-like skin pathology in multiple experimental models under both acute and chronic perturbations and in preventing both local and consequential systemic effects.

### Identification of downstream targets of Ovol1 in AD-like skin

**Fig. 4:**
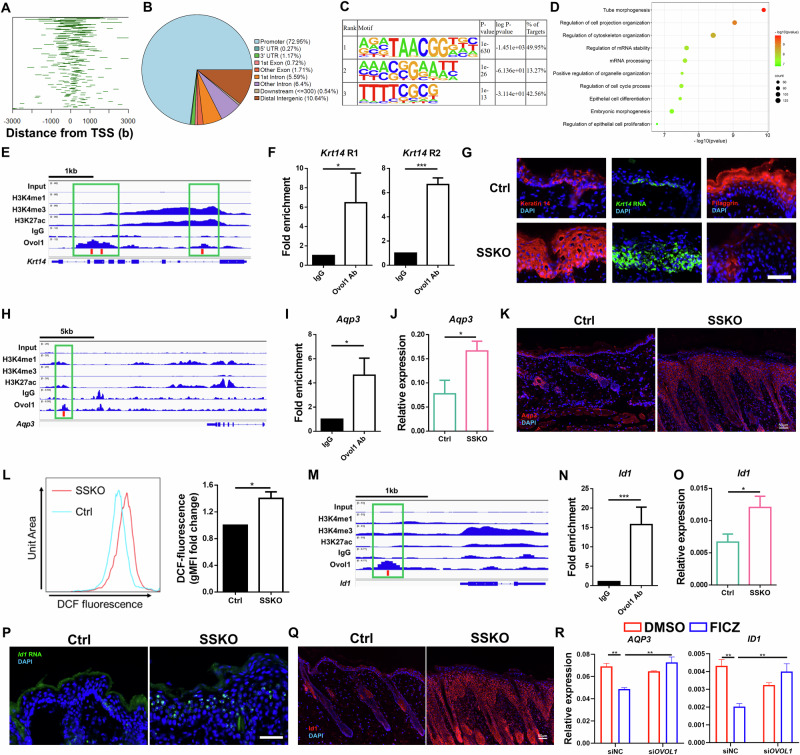
Identification of direct targets of Ovol1. **A** Line plots of the distance between the Ovol1 ChIP-seq peaks and the TSS. **B** Pie charts depicting annotated genomic features of the called ChIP-seq peaks. **C** Homer motif analysis showing the predominance of the Ovol consensus motif in the ChIP-seq peaks. **D** Metascape analysis of all the genes associated with peaks for Ovol1. **E** Genome browser track for the indicated ChIP-seq signals across the *Krt14* locus. **F** ChIP‒qPCR results of *Krt14* in mouse keratinocytes treated with Ca^2+^ (1.8 mM) for 24 h. The IgG control values were normalized to 1. Data are presented as the means ± SEMs. The results are summarized from 3 independent experiments. **G** Representative immunofluorescence images of keratin 14 protein, filaggrin protein, and RNAScope data of *Krt14* mRNA in the lesional skin of HDM/SEB-treated Ctrl and SSKO mice on day 11. n = 3 mice per group. Scale bar = 50 μm. **H** Genome browser track for the indicated ChIP-seq signals across the *Aqp3* locus. **I** ChIP‒qPCR results for *Aqp3*. See **F** for details. **J** RT‒qPCR results showing *Aqp3* expression in the whole skin of HDM/SEB-treated Ctrl (n = 6) and SSKO (n = 5) mice on day 11. Data are presented as the means ± SEMs. **K** Representative immunofluorescence images of Aqp3 in the lesional skin of HDM/SEB-treated Ctrl and SSKO mice on day 11. n = 4 mice per group. Scale bar = 50 μm. **L** Geometric mean fluorescence intensity (gMFI) values of DCF fluorescence in epidermal keratinocytes from HDM/SEB-treated Ctrl and SSKO mice on day 11. The values of the Ctrl mice were normalized to 1. n = 3 mice per group. **M** Genome browser track for the indicated ChIP-seq signals across the *Id1* locus. The red bars in **E**, **H**, and **M** indicate the presence of the Ovol1 binding motif (CCGTTA), and the green boxes highlight the Ovol1-bound regions. **N** ChIP‒qPCR results for *Id1*. See **F** for details. **O** RT‒qPCR results for *Id1*. See **J** for details. **P** Representative RNAScope data of *Id1* mRNA in the lesional skin of HDM/SEB-treated Ctrl and SSKO mice on day 11. n = 3 mice per group. Scale bar = 50 μm. **Q** Representative immunofluorescence images of Id1 in the lesional skin of HDM/SEB-treated Ctrl and SSKO mice on day 11. n = 4 mice per group. Scale bar = 50 μm. **R** RT‒qPCR results of the indicated genes in DMSO- or FICZ-treated NHEKs with or without *OVOL1* knockdown under M6 treatment conditions. The results are summarized from 3 independent experiments. DAPI in **G**, **K**, **P** and **Q** stains the nuclei. Data are presented as the means ± SEMs. *p < 0.05, **p < 0.01, ***p < 0.001. *p* values were calculated via 2-tailed unpaired Student’s *t* test (**F**, **I**, **J**, **L**, **N** and **O**) or *t*wo-way ANOVA (**R**)

To probe how Ovol1 protects against AD-like barrier dysregulation and skin pathology, we sought to identify its direct targets via ChIP-seq in primary mouse keratinocytes [32]. Using MACS2 broad peak calling, a total of 1109 Ovol1-bound peaks were identified that passed a significance threshold (*q* value < 0.05) (Supplementary Table S21). A large fraction (73%) of the peaks were found to reside in gene promoter regions within 1 kb of the transcription start site (TSS) (Fig. 4A, B). Substantial binding to distal intergenic sequences (10.6%) was also observed (Fig. 4B). Homer analysis revealed that ~50% of the Ovol1-bound loci contain at least one consensus binding motif (CCGTTA) (Fig. 4C), which is identical to the consensus binding sequence previously identified for recombinant Ovol1 [29]. Interestingly, 42.6% of the Ovol1-bound loci contain TTTTCGCG (Fig. 4C), a consensus motif for E2F transcription factor binding, suggesting that Ovol1 may also be recruited to a subset of its cognate promoters through binding to E2F sequences and/or through E2F/Rb complexes [48]. Metascape analysis of the Ovol1-bound genes revealed “tube morphogenesis”, “regulation of cell projection organization”, “regulation of cytoskeleton organization”, “regulation of mRNA stability”, “positive regulation of organelle organization”, and “regulation of the cell cycle process” as the top terms (Fig. 4D; Supplementary Table S22).

When we delved deeper into Ovol1 regulation of the cytoskeleton, we noted its direct binding to several keratin genes, including *Krt5*, *Krt14*, and *Krt10* (Supplementary Table S21). *Krt14* encodes keratin 14, an epidermal basal (stem/progenitor) cell-specific keratin that not only supports basal cell structure but also regulates keratinocyte proliferation and differentiation [49]. Our ChIP-seq results revealed Ovol1 binding at two regions in the *Krt14* locus with different extents of H3K27ac, H3K4me1 and H3K4me3, which are histone modifications of active promoters/enhancers [50–53] (Fig. 4E). This binding was confirmed via ChIP‒qPCR (Fig. 4F). Importantly, the expression of both *Krt14* mRNA and keratin 14 protein was considerably greater in the epidermis of HDM/SEB-treated SSKO mice than in that of control mice (Fig. 4G). This increase, together with the dramatically reduced expression of the terminal differentiation marker filaggrin in the suprabasal compartment of the SSKO epidermis (Fig. 4G), suggests that *Ovol1*-deficient keratinocytes are arrested in a progenitor cell/early differentiation state at least in part because of the loss of direct Ovol1-mediated repression of *Krt14* expression.

Next, we cross-examined the Ovol1 ChIP-seq hits with genes whose expression was upregulated upon *OVOL1* knockdown (Supplementary Fig. S1D, E) or *Ovol1* deficiency. This comparison identified 17 Ovol1-bound genes (e.g., *Dmwd*, *Fosb*, *Ganab*, and *Hic1*) whose human homologs presented increased expression in OVOL1-depleted NHEKs (Supplementary Fig. S4A) and 39 Ovol1-bound genes (e.g., *Aqp3*, *Id1*, *Slpi*, and *Stra6*) whose expression was increased in *Ovol1*-deficient mouse epidermis (Supplementary Fig. S4B). The terms “organic hydroxyl compound transport” (*Aqp3*, *Slc16a1* and *Stra6*) and “positive regulation of supramolecular fiber organization” (*Id1*, *Psrc1* and *Efemp2*) were enriched in the 39 mouse genes (Supplementary Fig. S4C). Elevated expression of *AQP3* and *ID1* upon *OVOL1* knockdown was also observed in NHEKs under suboptimal culture conditions (Supplementary Fig. S4D), leading us to focus on *Aqp3* and *Id1* as candidate Ovol1 targets in AD-like mouse skin inflammation.

A putative enhancer ~13 kb downstream of the *Aqp3* gene was found to contain an Ovol1-binding consensus, and both ChIP-seq and ChIP‒qPCR results revealed that Ovol1 binds to this site in mouse keratinocytes (Fig. 4H, I). RT‒qPCR analysis revealed a significant increase in *Aqp3* mRNA levels, whereas immunofluorescence staining revealed increased Aqp3 protein expression in the lesional skin of HDM/SEB-treated SSKO mice compared with that in the control skin (Fig. 4J, K). Since Aqp3 is known to promote H_2_O_2_ accumulation during skin inflammation [54], we compared H_2_O_2_ levels in the keratinocytes of HDM/SEB-treated SSKO and control mice and found that H_2_O_2_ levels were significantly elevated in the former (Fig. 4L).

*Id1* is a known Ovol1 target in trophoblast cells and has been shown to promote keratinocyte proliferation [55–57]. Our ChIP-seq and ChIP‒qPCR results revealed that Ovol1 binds to a putative upstream enhancer of *Id1* in mouse keratinocytes and that this enhancer contains an Ovol1-binding consensus sequence (Fig. 4M, N). RT‒qPCR/RNAScope and immunofluorescence staining revealed increased *Id1* mRNA levels and expanded protein expression in the lesional skin epidermis of HDM/SEB-treated SSKO mice compared with their control counterparts (Fig. 4O‒Q). Additionally, *OVOL1* knockdown led to a significant increase in the expression of both *AQP3* and *ID1* in FICZ-treated, but not DMSO-treated, NHEKs (Supplementary Fig. S4E). Moreover, FICZ-induced inhibition of *AQP3* and *ID1* mRNA expression in NHEKs treated with an inflammatory cytokine cocktail (containing IL-4, IL-17A, IL-22, oncostatin M, IL-1α, and TNF-α; see below) was alleviated by the depletion of *OVOL1* (Fig. 4R). Together, these data show that Ovol1 plays a direct role in regulating genes associated with the cytoskeleton, reactive oxygen species, and cellular proliferation in AD-like skin. Additionally, they suggest the presence of a conserved AhR-Ovol1-Aqp3/Id1 regulatory axis.

### Id1 regulates epidermal barrier integrity and neutrophil accumulation downstream of *Ovol1* in AD-like skin

**Fig. 5:**
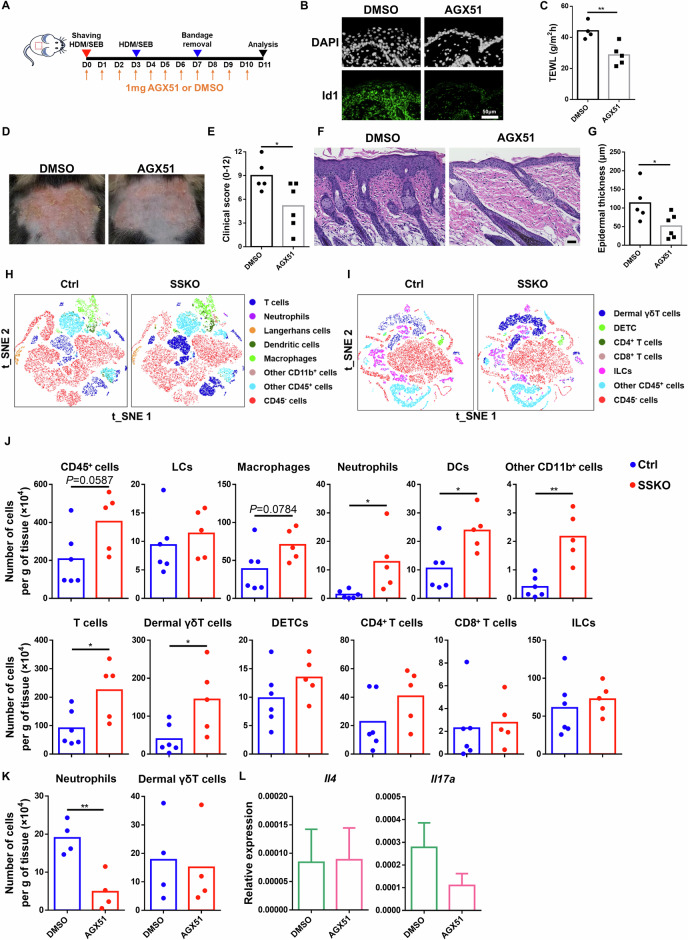
Inhibition of Id1 rescues AD-like symptoms, barrier disruption, and neutrophil accumulation in HDM/SEB-treated SSKO mice. **A** Experimental design for AGX51 treatment in **B**–**G**, **K**–**L**. DMSO was used as a vehicle control. **B** Representative immunofluorescence images of Id1 in the lesional skin of HDM/SEB-treated WT mice on day 11. DAPI was used to stain the nuclei. n = 5 mice per group. Scale bar = 50 μm. **C** TEWL values of DMSO- or AGX51-treated SSKO mice on day 11. n = 4–5 mice per group. **D** Representative photographs of DMSO- or AGX51-treated SSKO mice on day 11. **E** Clinical scores of the DMSO- or AGX51-treated SSKO mice on day 11. n = 5–6 mice per group. **F** Representative skin histology (H&E staining) on day 11. Scale bar = 50 μm. **G** Quantification of epidermal thickness. n = 5–6 mice per group. **H**–**J** Flow cytometry analysis of the back skin of HDM/SEB-treated Ctrl and SSKO mice on day 11. t-SNE visualization of the indicated cell types (**H**, **I**) and quantification of the number of the indicated immune cell types per gram of skin tissue (**J**) are shown. n = 6 for the Ctrl; n = 5 for the SSKO. **K** Quantification of the indicated immune cell types per gram of skin tissue in DMSO- or AGX51-treated SSKO mice on day 11. n = 4 mice per group. **L** RT‒qPCR results showing *Il4* and *Il17a* expression in whole skin. Data are presented as the means ± SEMs. n = 4 mice per group. *p < 0.05, **p < 0.01. *p* values were calculated via 2-tailed unpaired Student’s *t* test

To determine whether aberrantly enhanced *Id1* expression contributes to the disrupted skin barrier and pathology of SSKO mice, we utilized AGX51, a small chemical inhibitor that induces Id1 protein degradation [58] (Fig. 5A, B, Supplementary Fig. S5A). Compared with the DMSO vehicle control, i.p. administration of AGX51 significantly reduced TEWL and epidermal thickness and improved the AD-like clinical score in HDM/SEB-treated SSKO mice (Fig. 5A, C–G, Supplementary Fig. S5B, C). AGX51 treatment also decreased the TEWL and epidermal thickness in the HDM/SEB-treated WT mice (Supplementary Fig. S5D–F). These results show that *Id1* is a functionally important target of Ovol1 in AD-associated epidermal barrier defects and skin pathology.

To gain further insight into the immunological responses of the skin to *Ovol1* deletion-induced AD-associated epidermal dysregulation, we conducted comprehensive immune cell profiling of control and SSKO skin during both homeostasis and AD-like inflammation. Langerhans cells (LCs) and dendritic epidermal T cells (DETCs) are immune cell types that reside within the mouse epidermis [59], and their relative abundance per total immune cells (CD45^+^) was not altered in the SSKO epidermis during homeostasis (Supplementary Fig. S6A–C). Similarly, the relative abundance of dermal T-cell subsets was unaffected in the SSKO mice under homeostatic conditions (Supplementary Fig. S6D–F). Upon stimulation with HDM and SEB, the SSKO skin contained significantly greater numbers of neutrophils, dendritic cells (DCs), CD11b^+^ cells, total T cells, and dermal γδT cells than did the control skin, whereas the numbers of LCs, DETCs, macrophages, CD4^+^ T cells, CD8^+^ T cells, and innate lymphoid cells (ILCs) were not significantly affected (Fig. 5H–J, Supplementary Fig. S7A–D). Intriguingly, dermal γδT cells were still increased in SSKO mice treated with 3 rounds of HDMs or SEB, whereas neutrophils and DCs were no longer significantly different from those in control mice at this time (Supplementary Fig. S7E–I).

Next, we examined the impact of Id1 inhibition on immune cell profiles in AD-like skin. Flow cytometry analysis revealed that AGX51 treatment significantly reduced the abundance of neutrophils, whereas dermal γδT and other immune cells remained unaffected in HDM/SEB-treated SSKO and WT mice (Fig. 5K, Supplementary Fig. S7J–M). Moreover, the expression of the type 2 and 3 cytokines *Il4* and *Il17a*, respectively, was not significantly altered by Id1 inhibition (Fig. 5L). These data identify a specific role for Id1 in regulating neutrophil accumulation downstream of Ovol1 in AD-like skin.

### IL-1 signaling promotes dermal γδT accumulation and pathology downstream of barrier dysregulation in AD-like SSKO skin

**Fig. 6:**
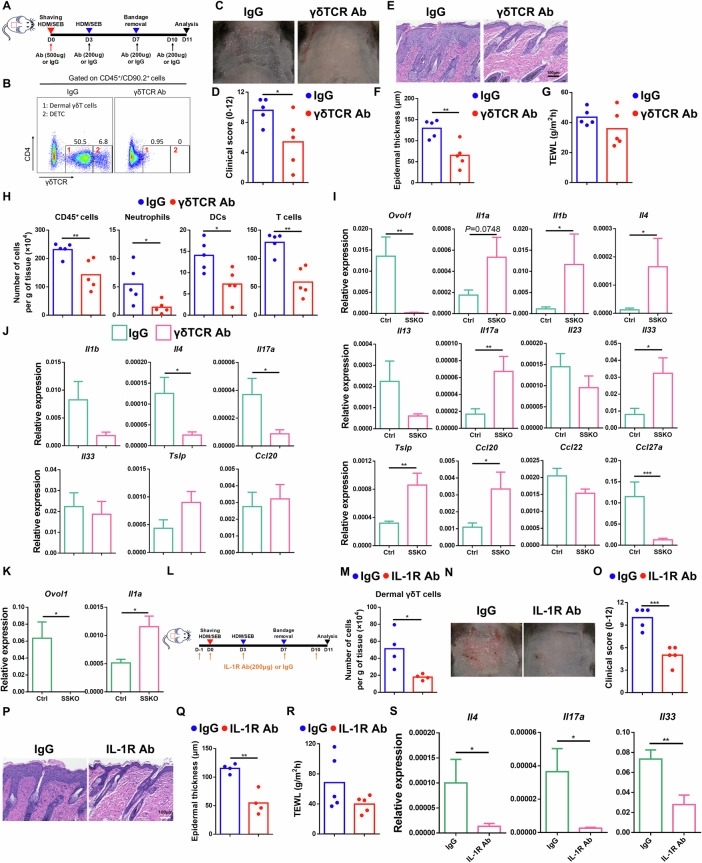
Effects of dermal γδT blockade and IL-1 signaling inhibition on skin inflammation in HDM/SEB-treated SSKO mice. **A** Experimental design for γδTCR antibody (Ab) treatment. IgG was used as a control. **B** Representative flow cytometry plots for dermal γδT cells (1) and DETCs (2) among CD45^+^CD90.2^+^ cells. The numbers in the plots represent the relative abundance of the indicated cell populations. **C** Representative images of IgG- or γδTCR Ab-treated SSKO mice on day 11. **D** Clinical scores of IgG- or γδTCR Ab-treated SSKO mice on day 11. n = 5 mice per group. **E** Representative skin histology (H&E staining) on day 11. Scale bar = 100 μm. **F** Quantification of epidermal thickness. n = 5 mice per group. **G** TEWL values of IgG- or γδTCR Ab-treated SSKO mice on day 11. n = 5 mice per group. **H** Quantification of the number of the indicated cell types per gram of skin tissue in IgG- or γδTCR Ab-treated SSKO mice on day 11. See the legend of Fig. 5 for additional details. n = 5 mice per group. **I**, **J** RT‒qPCR results of the indicated genes in the whole skin of HDM/SEB-treated Ctrl (n = 6) and SSKO (n = 5) mice (**I**) or of IgG- or γδTCR Ab-treated SSKO mice (n = 5 mice per group) on day 11 (**J**). **K** RT‒qPCR results of *Ovol1* and *Il1a* expression in epidermal cells of HDM/SEB-treated mice on day 7. n = 3 mice per group. **L** Experimental design for IL-1R Ab treatment (M-S). The samples were harvested on day 11 after treatment for downstream analysis. IgG was used as a control. **M** Flow cytometry analysis of dermal γδT cells in the back skin of IgG- or IL-1R Ab-treated SSKO mice. See the legend of Fig. 5 for additional details. n = 4 mice per group. **N** Representative images of IgG- or IL-1R Ab-treated SSKO mice. **O** Clinical scores of IgG- or IL-1R Ab-treated SSKO mice. n = 5 mice per group. **P** Representative skin histology (H&E staining). Scale bar = 100 μm. **Q** Quantification of epidermal thickness in IgG- or IL-1R Ab-treated SSKO mice. n = 4 mice per group. **R** TEWL values of IgG- or IL-1R Ab-treated SSKO mice. n = 5 mice per group. **S** RT‒qPCR results showing *Il4*, *Il17a*, and *Il33* expression in whole skin. n = 5 mice per group. Data are presented as the means ± SEMs. * *p* < 0.05, ** *p* < 0.01, *** *p* < 0.001. *p* values were calculated via 2-tailed unpaired Student’s *t* test or the Mann‒Whitney U tes*t*

The dramatic and persistent increase in dermal γδT cells in AD-like SSKO skin led us to investigate whether these cells function downstream of *Ovol1* deficiency-induced barrier dysregulation to promote exacerbated inflammation and pathology. These γδTCR-expressing innate T cells are integral components of the local immunosurveillance program in the skin; they typically serve as kick-starters of inflammation and are often the main producers of IL-17 in various models of inflammatory diseases [60–64]. However, their roles in AD-like skin inflammation appear complex, with an anti-inflammatory effect being suggested [65, 66]. We administered a γδTCR antibody to HDM/SEB-treated mice to functionally block γδT cells [67–69] (Fig. 6A). As expected, the γδTCR antibody nearly completely prevented flow cytometry detection of dermal γδT cells and DETCs (Fig. 6B). Importantly, this antibody treatment significantly alleviated AD-like skin phenotypes in SSKO mice, as indicated by the clinical score and epidermal and dermal thickness (Fig. 6C–F, Supplementary Fig. S8A, B), but did not significantly restore epidermal barrier function (Fig. 6G). Similar effects were observed in the AD-like skin of WT mice (Supplementary Fig. S8C–E). Flow cytometry analysis revealed that the numbers of total immune cells, neutrophils, DCs, and T cells in AD-like SSKO skin were significantly reduced by γδTCR antibody treatment (Fig. 6H, Supplementary Fig. S8F–H). These data underscore the critical role of γδT cells (presumed to be dermal γδT cells because of their elevated accumulation in SSKO skin) in orchestrating both innate and adaptive immunity downstream of epidermal/barrier dysregulation in AD-like skin.

To define the molecular consequences of γδT blockade in AD-like skin, we first comprehensively assessed cytokine/chemokine changes in HDM/SEB-treated control and SSKO mice. The expression of *Il4*, *Il17a*, *Il33*, *Tslp* and *Ccl20* was significantly upregulated in SSKO skin, whereas the expression of *Il13*, *Il23, Ccl22* and *Ccl27a* was either unaffected or downregulated (Fig. 6I). These data suggest that some, though not all, molecular aspects of type 2 (*Il4, Il33* and *Tslp*) and type 3 (*Il17a* and *Ccl20*) immunity are enhanced in AD-like SSKO skin. RT‒qPCR analysis revealed significantly decreased *Il4* and *Il17a* expression in the SSKO skin lesions following γδTCR antibody treatment (Fig. 6J), suggesting that dermal γδT cells contribute to the potentiation of type 2 (Th2) immune responses in AD-like SSKO skin.

Next, we investigated how epidermal dysregulation in SSKO mice elicits a dermal γδT response. Barrier disruption can result in elevated expression of alarmins such as IL-1α [31, 70, 71]. Indeed, we observed upregulation of both *Il1a* and *Il1b* expression in the lesional skin of SSKO mice (Fig. 6I), with a significant increase in *Il1a* expression specifically detected within the epidermis (Fig. 6K). To test whether these changes mediate excessive dermal γδT cell accumulation in AD-like skin, we i.p. administered an IL-1R antibody to HDM/SEB-treated SSKO and WT mice to block IL-1 signaling (Fig. 6L). Compared with the IgG control, this antibody treatment resulted in an ~2.5-fold reduction in the number of dermal γδ T cells in AD-like SSKO skin, whereas other immune cell types were not significantly affected (Fig. 6M, Supplementary Fig. S9A–E). Furthermore, AD-like skin defects in HDM/SEB-treated SSKO mice were dramatically alleviated by the anti-IL-1R antibody, resulting in improved clinical scores and reduced epidermal thickness (Fig. 6N–Q, Supplementary Fig. S9F). IL-1R antibody treatment also decreased dermal γδ T cell infiltration and epidermal hyperplasia in WT mice (Supplementary Fig. S9G–I). However, TEWL and dermal thickness were not significantly affected (Fig. 6R, Supplementary Fig. S9J, K). At the molecular level, the expression of *Il4, Il17a*, and *Il33*, but not that of *Tslp*, *Ccl20* or *Ccl27a*, was dramatically lower in the skin lesions of the IL-1R antibody-treated SSKO mice than in those of their IgG-treated SSKO littermates (Fig. 6S, Supplementary Fig. S9L). Taken together, these data show that IL-1 signaling functions downstream of barrier dysregulation to promote dermal γδT accumulation and the associated AD-like pathology.

### Relevance of OVOL1 and ID1 functions in human AD skin

**Fig. 7:**
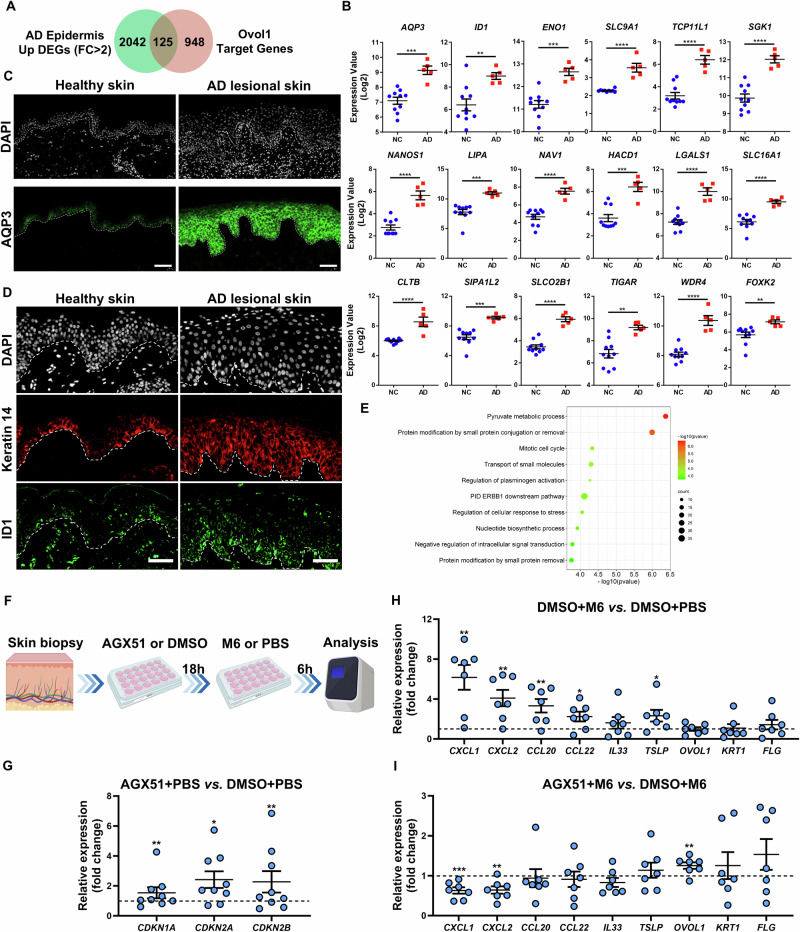
OVOL1 target gene expression and function in human AD skin. **A** Venn diagram of ChIP-seq-identified Ovol1 target genes and genes upregulated in the human AD epidermis. The AD data were derived from published RNA-seq results (GSE120721). Gene numbers are indicated in the Venn diagram. **B** Expression of a select number of overlapping genes from **A** in the epidermis of normal (n = 10) and AD lesional (n = 5) skin. **C** Immunostaining of the AQP3 protein in healthy and AD lesional skin. n = 10 samples per group. Scale bar = 100 μm. **D** Immunostaining of keratin 14 and ID1 proteins in healthy and AD lesional skin. n = 10 samples per group. Scale bar = 50 μm. The dashed lines in **C** and **D** indicate the basement membrane, and DAPI was used to stain the nuclei. **E** Ingenuity pathway analysis of the 125 overlapping genes in **A**. **F** Experimental design for ex vivo human skin biopsy stimulation in **G**–**I**. **G** RT‒qPCR results of the indicated genes in AGX51- or DMSO-treated human skin biopsies. n = 9 samples per group. **H** RT‒qPCR results of the indicated genes in M6- or PBS-treated human skin biopsies. n = 7 samples per group. **I** RT‒qPCR results of the indicated genes in AGX51- or DMSO-treated human skin biopsies subjected to M6 treatment. n = 7 samples per group. **p < 0.01, ***p < 0.001, ****p < 0.0001. *p* values were calculated via 2-tailed unpaired Student’s *t* test

The functional significance of Ovol1 in suppressing AD-like mouse skin inflammation prompted us to investigate whether the expression of its target genes is altered in human AD skin. Cross-analysis of our Ovol1 ChIP-seq data with published RNA-seq data from the human AD epidermis [43] led to the identification of human homologs of 125 Ovol1 target genes, which included *AQP3* and *ID1*, whose expression was significantly increased in the AD epidermis (Fig. 7A, B, Supplementary Fig. S10A, Supplementary Table S23). Immunostaining experiments confirmed the dramatically increased/expanded expression of the AQP3 and ID1 proteins in AD epidermal keratinocytes (Fig. 7C, D). Thus, *Ovol1* deficiency-induced upregulation of *Aqp3* and *Id1* in mouse AD-like skin is likely mirrored in human AD skin. Metascape analysis of the 125 genes revealed “pyruvate metabolic process”, “protein modification by small protein conjugation or removal”, “mitotic cell cycle”, and “transport of small molecules” as the top terms (Fig. 7E, Supplementary Table S24). Pyruvate metabolism is known to promote keratinocyte proliferation [72]. The specific upregulated genes associated with pyruvate metabolism are *ENO1*, *SLC9A1, FOXK2*, *LIPA*, *SLC16A1*, *TIGAR*, *ATF3*, *PPP4R3B*, *PEX13*, *HACD1*, *PANK2*, *GLDC*, *NDUFAF1*, *LANCL2*, *PRPS1*, *LPCAT3*, *DTYMK*, *PATL1*, *CD44* and *PFKP* (Fig. 7B), some of which encode metabolic enzymes or transporters or transcription factors relevant to glycolysis [50, 73–77], indicating a potential role of OVOL1 in regulating cellular metabolism in AD skin.

As proof-of-principle validation of the functionality of OVOL1 targets, we tested the effect of the ID1 inhibitor AGX51 on explant cultures derived from skin biopsies of healthy individuals (Fig. 7F). We found that AGX51 significantly increased the expression of several canonical ID1 target genes, including the cell cycle regulators *CDKN1A*, *CDKN2A*, and *CDKN2B* [51] (Fig. 7G). We also treated explants with a cytokine cocktail comprising IL-17A, IL-22, oncostatin M, IL-1α, and TNF-α, which are known to stimulate keratinocytes to produce various skin inflammatory cytokines and chemokines [52]. This approach, augmented with the addition of IL-4, resulting in a formulation named M6, was employed to establish an ex vivo human AD-like skin model (Fig. 7F). While M6 treatment resulted in elevated expression of *CXCL1*, *CXCL2*, *CCL20*, *CCL22* and *TSLP* (Fig. 7H), the addition of AGX51 dampened this effect, as evidenced by the significantly reduced expression of the neutrophil chemoattractants *CXCL1* and *CXCL2* (Fig. 7I). Intriguingly, AGX51 treatment also slightly but significantly increased *OVOL1* expression in M6-treated skin explants, which may reflect altered differentiation or possible feedback regulation. Taken together, our data suggest that elevated activity of an OVOL1 target, ID1, has the capacity to functionally contribute to human skin inflammation through augmenting neutrophil accumulation.

To investigate whether the AhR-OVOL1-ID1 axis is specific to AD or represents a broader mechanism in inflammatory skin diseases, we analyzed biopsy samples from patients with psoriasis. Our analysis revealed decreased expression levels of the AhR protein and *OVOL1* mRNA, along with increased levels of the AQP3 and ID1 proteins, in the skin lesions of these patients (Supplementary Fig. S10B–E), mirroring findings observed in human AD skin lesions. These data suggest that dysregulation of the AhR-OVOL1-ID1 axis may be a shared feature of AD and psoriasis.

## Discussion

**Fig. 8:**
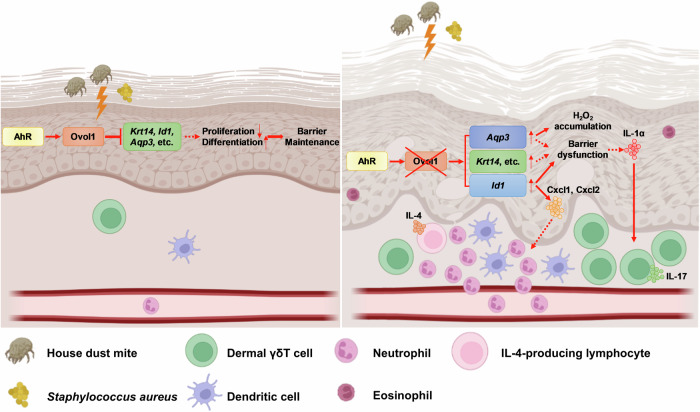
Working model of Ovol1 regulation of epidermal and immune homeostasis during AD-like skin inflammation. The solid lines indicate regulations for which we present evidence in this study. The dashed lines indicate regulations reported in the literature. Thin upward arrows indicate aberrant upregulation when *Ovol1* is ablated. This figure was created via BioRender.com

Our study highlights the critical importance of regulating keratinocyte proliferation and differentiation for a resilient skin barrier against external stressors. Keratinocytes play a key role not only in forming a physical barrier but also in secreting signaling molecules that influence immune cell activity, contributing to inflammatory skin conditions such as AD [2, 53, 78]. We identified an *OVOL1/Ovol1*-directed gene expression program that operates downstream of AhR signaling in keratinocytes. This program serves to suppress proliferation, regulate metabolism, promote terminal differentiation, enhance barrier integrity, and modulate the immune response against allergens and pathogens implicated in AD (Fig. 8). These findings reveal an intricate transcriptional regulatory program that coordinates epidermal homeostasis and immune function at the skin barrier, safeguarding against AD-inducing assaults.

Our discovery of *OVOL1*/*Ovol1* as a direct target and pivotal mediator of the barrier-protective and anti-inflammatory functions of AhR lays the groundwork for developing therapeutics targeting OVOL1-associated cellular and molecular components. Such targeted therapies hold promise for effectively preventing and treating inflammatory skin diseases such as AD and psoriasis while minimizing adverse effects associated with AhR activation in immune cells. For example, the activation of AhR in CD4^+^ T cells can drive differentiation toward the Th17 and Th22 subtypes [79], which may inadvertently trigger skin inflammation in patients suffering from AD or psoriasis.

Our research also highlights previously unrecognized players in skin inflammation and AD pathogenesis. Specifically, our studies in both humans and mice suggest that ID1/Id1, a direct target of OVOL1/Ovol1 transcriptional repression in keratinocytes, plays a role in promoting AD-associated epidermal hyperplasia, barrier disruption, and clinical symptoms. While the involvement of ID1 in keratinocyte proliferation is known [57], its role in skin inflammation has not been previously reported. The effects of ID1/Id1 inhibition on decreasing the expression of neutrophil chemoattractants in human skin explants and neutrophil accumulation in *Ovol1*-deficient AD-like mouse skin suggest that ID1/Id1 specifically functions in augmenting a neutrophilic response.

Our work suggests that dermal γδT cells are significant contributors to AD-like skin inflammation in mice. We found that IL-1 signaling plays a crucial role in amplifying the dermal γδT cell response in AD-like skin downstream of barrier dysregulation. Both functional blockade of γδT cells and inhibition of IL-1 signaling led to reduced expression of not only *Il17* but also the type 2 cytokine *Il4*, suggesting a role for the IL-1/γδT axis in orchestrating type 2 immunity. Interestingly, γδTCR signaling is required not only for maximal neutrophil infiltration but also for DC expansion in AD-like SSKO skin. Given that DCs can recruit both type 2 (Th2) and type 3 (Th17) T cells to inflamed skin [2], γδT-facilitated DC accumulation may be a mechanism by which Th2 cells are recruited to AD-like SSKO skin. Given the low frequency of peripheral γδT cells in human skin [80], the relevance of our mouse γδT findings to human AD remains uncertain. Nonetheless, understanding how the epidermis regulates dermal γδT cells to influence type 2 and type 3 immunity sheds light on the missing links between epidermal barrier dysfunction and adaptive immune responses.

In conclusion, our study identified an AhR-Ovol1-Id1 regulatory axis in keratinocytes that governs both epidermal and immune homeostasis against AD-inducing allergens and pathogens while also implicating a similar mechanism in psoriasis. While not all molecular details of this axis are identical between mice and humans, the core regulatory mechanisms and functions appear to be conserved. These findings pave the way for future studies to delve deeper into the role of this axis in human AD and psoriasis.

## Materials and methods

### Cell culture and transfection

NHEKs were cultured in keratinocyte serum-free medium (Gibco, Cat# 17005042) per the manufacturer’s protocol and used within passages 3--6 for experiments. Scrambled *AHR*- or *OVOL1*-specific siRNAs (generated by Huzhou Hippo Biotechnology Co., Ltd.) were transfected into NHEKs via Lipofectamine RNAiMAX Transfection Reagent (Thermo Fisher Scientific, Cat# 13778075) according to the manufacturer’s protocol.

### Mice

*K14-*Cre;*Ovol1*^+/−^ (C57BL/6 background), *Ovol1*^−/−^ (CD1 background), and *Ovol1*^flox/flox^ (C57BL/6 background) mice were generated as described previously [30, 31]. *Ahr*^flox/flox^ mice were generated via CRISPR/Cas9 technology, with loxP sites strategically inserted into exon 5 of the *Ahr* gene. The *Ahr*^flox/flox^ mice were bred with *K14*-Cre mice to obtain mice with conditional knockout of the *Ahr* gene, specifically in keratinocytes. Sex- and weight-matched control and mutant littermates were used for all experiments. Both male and female 7–10-week-old mice were used. All the animal studies were approved and abided by the regulatory guidelines of the Institutional Animal Care and Use Committee of the University of California, Irvine.

### AD models

For the mouse AD model, the mice were epicutaneously sensitized with HDMs and SEB as described previously [33] or treated with MC903 [46, 47].

In brief, a solution of 10 μg of HDM extract (Stallergen Greer) and 500 ng of SEB (List Biological Laboratories) in PBS was applied to a 1 cm^2^ gauze pad placed on the shaved skin and occluded with a Tegaderm Transparent Dressing (3 M Healthcare). Three days later, the gauze pads were replaced. Four days later, the dressings were removed, and the mice were kept without treatment for 4 days and then sacrificed for analysis. For experiments with three rounds of treatment, the mice received this “3 + 4-d” pattern of treatment twice more and were sacrificed two days after the last cycle of treatment. TEWL was measured on shaved mouse back skin via a Delfin VapoMeter (SWL4400). The TEWL values are output as g/m^2^h. To quantify AD-like symptoms, skin eruption, scaling, bleeding and redness were blindly scored independently by one or more investigators on a scale ranging from 0 (none) to 3 (most severe). The cumulative score served as a measure of the severity of clinical signs (score 0–12).

Two regimens were employed for the MC903-induced AD model. For *Ovol1*^−/−^ mice, we topically applied 4 nmol of MC903 (Sigma, St. Louis, Missouri) or ethanol control to the mouse ears once daily for 14 consecutive days. The mice were sacrificed on day 15. For the SSKO mice, we topically applied 1.125 nmol of MC903 or ethanol to the mouse ears once daily for 5 consecutive days; the mice were then kept without treatment for 2 days, followed by topical application of 1.125 nmol of MC903 once daily for 3 more days. The mice were sacrificed on day 11. Ear thickness was measured via a caliper.

For the ex vivo human AD model, skin tissues (approximately 0.25 or 0.5 cm^2^) from 9 healthy individuals were obtained via excisional biopsy during plastic surgery and divided into 2 or 4 equal parts. The tissues were cultured in DMEM (Gibco, Cat# 11965092) containing 10% FBS and treated with AGX51 (100 μM) or DMSO for 18 hours, followed by treatment with M6 cytokine cocktail or PBS for another 6 hours. The M6 cocktail contains IL-4 (10 ng/ml, Peprotech, Cat# 200--04), IL-17A (10 ng/ml, Peprotech, Cat# 200--17), IL-22 (10 ng/ml, Peprotech, Cat# 200--22), oncostatin M (10 ng/ml, Peprotech, Cat# 300--10), IL-1α (10 ng/ml, Peprotech, Cat# 200--01 A), and TNF-α (10 ng/ml, Peprotech, Cat# 300--01 A). After M6 treatment, the skin tissues were collected and stored in liquid nitrogen until further analysis.

### In vivo FICZ, antibody, and AGX51 treatment

For FICZ experiments, the mice were i.p. injected with 100 μg/kg FICZ (MedChemExpress, Cat# HY-12451) or DMSO in 100 μl of corn oil once daily from day 0 to day 10. For γδTCR blockade, the mice were i.p. injected with 500 μg γδTCR antibody (BioLegend, Cat# 107517, Clone UC7-13D5) or IgG (BioLegend, Cat# 400959, Clone HTK888) on day 0, followed by 200 μg at days 3, 7 and 10 of HDM/SEB treatment. For IL-1R blocking, the mice were i.p. injected with 200 μg of an anti-IL-1R antibody (BioXcell, Cat# BE0256; Clone JAMA-147) or IgG (BioXcell, Cat# BE0091) at days -1, 0, 3, 7 and 10 of HDM/SEB treatment. For the AGX51 experiments, the mice were i.p. injected with 1 mg of AGX51 (MedChemExpress, Cat# HY- 129241) or DMSO in 100 μl of corn oil once daily from day 0 to day 10.

### Histology, indirect immunofluorescence, and RNAScope

Sections from paraformaldehyde-fixed, paraffin-embedded back skin were stained with hematoxylin and eosin (H&E) as previously described [30]. Epidermal and dermal thickness was measured via ImageJ. For indirect immunofluorescence of keratin 14, filaggrin, Ki67 and Ly6G, mouse back skin tissues were freshly frozen in optimum cutting temperature (OCT) compound (Tissue-Tek) and stained with the appropriate antibodies as described previously [30]. The primary antibodies used were as follows: keratin 14 and filaggrin (rabbit or chicken, gifts from Julie Segre, National Institutes of Health, Bethesda), Ki67 (rabbit, Cell Signaling Technology, Cat# 9129, Clone D3B5), and Ly6G (rat, eBioscience, Cat# 16-9668-82, Clone 1A8). For indirect immunofluorescence of Aqp3, Id1, CD3, and GATA3, sections from paraformaldehyde-fixed, paraffin-embedded back skin were stained as previously described [81] with the appropriate antibodies. Antigen retrieval was performed by incubating slides in 0.01 M citrate buffer (pH 6.0) in a microwave at full power for 3–5 min. For immunostaining of AhR, sections from paraformaldehyde-fixed, paraffin-embedded back skin were stained via a three-color fluorescence kit (Recordbio Biological Technology, Cat# RC0086-23RM) based on the tyramide signal amplification technology according to the manufacturer’s instructions. Antigen retrieval was performed by incubating slides in 1 mM EDTA Antigen Retrieval Solution (pH 9.0) in a microwave at full power for 3–5 min. The primary antibodies used were as follows: Aqp3 (rabbit, Abcam, Cat# ab125219), Id1 (mouse, Santa Cruz Biotechnology, Cat# sc-133104, Clone B-8), CD3 (rabbit, Abcam, Cat# ab16669, Clone SP7), GATA3 (gift from J. Segre, National Institutes of Health, Bethesda) and AhR (rabbit, Proteintech, Cat# 28727-1-AP). RNAScope was performed as described previously [82] via a *Krt14* probe (ACD, Cat# 422521-C3).

### Patient sample analysis

Skin samples were obtained from 10 AD patients, 5 psoriasis patients and 10 healthy individuals by excisional biopsy. Sample acquisition, including skin biopsy, was approved by the Ethics Committee of Shanghai Tenth People’s Hospital and performed in accordance with the Declaration of Helsinki principles. Informed consent was obtained for all procedures.

Paraffin blocks of skin samples were used to obtain 4-μm sections, which were used for immunofluorescence and fluorescence in situ hybridization as previously described [65]. The antibodies used for immunofluorescence were anti-AhR (rabbit, Proteintech, Cat# 28727-1-AP), anti-cytokeratin 10 (rabbit, Servicebio, Cat# GB112105-100), anti-cytokeratin 14 (rabbit, Abcam, Cat# ab119695), anti-ID1 (mouse, Santa Cruz, Cat# sc-133104), and anti-aqp3 (rabbit, Servicebio, Cat# GB11395-100) antibodies. The immunostaining of AhR, AQP3, or costaining of keratin 14 and ID1 was performed via a three-color fluorescence kit (Recordbio Biological Technology, Cat# RC0086-23RM) based on tyramide signal amplification technology according to the manufacturer’s instructions. For maximum detection of *OVOL1* RNA, a 1:1:1:1 mixture of four different probes (Table S25) was used.

### Single-cell suspension and flow cytometry

A single-cell suspension of skin was prepared as described previously [30]. For cell surface staining, the cells were incubated with TruStain FcX™ Antibody (BioLegend, Cat# 101319) to block Fc receptors for 10 min at room temperature and incubated with antibodies for 30 min at 4 °C. For intracellular staining, the cells were incubated with a Zombie NIR™ Fixable Viability Kit (BioLegend, Cat# 423105) for 30 min at 4 °C before cell surface staining. After cell surface staining, the cells were fixed with IC fixation buffer (eBioscience, Cat# 00--8222--49) for 30 min at 4 °C and then stained with antibodies in permeabilization buffer (eBioscience, Cat# 00--8333--56) for 30 min at 4 °C. The surface-stained cells were incubated with SYTOX™ Blue Dead Cell Stain (Invitrogen, Cat# S34857) for 5 min before analysis. The cells were analyzed via a BD FACSAria Fusion Sorter. The data were analyzed via FlowJo v10.7.2.

### ChIP analysis

Mouse primary keratinocytes were treated with CaCl_2_ (1.8 mM) for 24 hours and then crosslinked in 1% formaldehyde. NHEKs were treated with FICZ (100 nM) for 3 h and then crosslinked in 1% formaldehyde. The ChIP assay was performed using an anti-OVOL1 antibody (rabbit, GeneTex, Cat# GTX55272), an anti-AhR antibody (rabbit, Cell Signaling Technology, Cat# 83200S), and a SimpleChIP® Enzymatic Chromatin IP Kit (agarose beads) (Cell Signaling Technology, Cat# 9002) according to the manufacturer’s instructions. The DNA was then purified, and RT‒qPCR was performed. Information on the gene-specific primers used is provided in Supplementary Table S25.

Ovol1 ChIP-seq data were analyzed as described previously [32]. Peaks were called with MACS2 broad peak calling [66]. Motifs were analyzed via HOMER v4.11 [83]. Public ChIP-seq BIGWIG data were downloaded from GEO datasets and visualized via IGV_2.10.2. The ChIP-seq data used were as follows: GSE86900 (Mouse H3K4me1, H3K4me3 and H3K27ac), GSE72455 (Mouse AhR), GSM733674 (NHEK H3K27ac), GSM733698 (NHEK H3K4me1), GSM733720 (NHEK H3K4me3) and GSM733740 (NHEK Input).

### Intracellular H_2_O_2_ measurement

The intracellular level of H_2_O_2_ was measured as described previously [54]. In brief, epidermal cells from mouse back skin were incubated with H2DCFDA (Invitrogen, Cat# D399) at a concentration of 10 μM at 37 °C for 30 min. The cells were then analyzed via a BD FACSAria Fusion Sorter, and the data were analyzed via FlowJo v10.7.2.

### Measurement of the serum levels of Cxcl2 and G-CSF

Custom Plex Assays (Eve Technologies) were used to detect the serum levels of Cxcl2 and G-CSF in MC903-treated mice according to the manufacturer’s instructions.

### Statistical analysis

Two-tailed unpaired Student’s *t* test was used to compare two groups if the data were normally distributed. The Mann‒Whitney U test was used to compare two groups if the data were not normally distributed. Two-way analysis of variance (ANOVA) was used to compare multiple groups. All analyses except single-cell RNA-seq data were performed via Prism 6 (GraphPad). *p* values of single-cell RNA-seq data were calculated via a t test followed by Benjamini‒Hochberg correction.

## Supplementary information

Supplemental Text

Supplemental Figures

Source Figure S5A

Supplemental Tables

## Data Availability

All data needed to evaluate the conclusions in the paper are presented in the paper or the Supplementary Materials. The requests for materials and correspondence should be addressed to X.D. (xdai@uci.edu) and Y.S. (shiyuling1973@tongji.edu.cn).
