# Supplementary material for: The AhR-Ovol1-Id1 regulatory axis in keratinocytes promotes epidermal and immune homeostasis in atopic dermatitis-like skin inflammation
Source: Cell Mol Immunol. 2025 Feb 13;22(3):300–15. doi: 10.1038/s41423-025-01264-z (PMC11868582; doi:10.1038/s41423-025-01264-z)
Supplement: Supplementary file 1 — Supplemental Text [file 41423_2025_1264_MOESM1_ESM.docx]

**An AhR-Ovol1-Id1 regulatory axis in keratinocytes promotes epidermal and immune homeostasis against atopic dermatitis-like skin inflammation**

Zeyu Chen^1,2,4,5^, Morgan Dragan^2,3^, Peng Sun^2,3^, Daniel Haensel^2,3^, Remy Vu^2,3^, Lian Cui^1,5^, Peiyao Zhu^1,5^, Nan Yang^1,5^, Yuling Shi^1,5,*^, and Xing Dai^2,3,6,7,*^

**SUPPLEMENTAL MATERIALS AND METHODS**

**RNA-seq and RT-qPCR**

For bulk RNA-seq and RT-qPCR, NHEKs were transfected with scrambled, *AHR*-specific or *OVOL1*–specific siRNA for 24 hours, and then treated with 100 nM FICZ (MedChemExpress, Cat# HY-12451) or 10% v/v of TSB (100 μl TSB to 900 μl medium, Sigma-Aldrich, Cat# 22090), which is typically used for microbial culture but lacks critical growth factors and other components required for mammalian cell growth (1), for another 24 hours. In some experiments, M6 was added in the last 6 hours of culture. Total RNA was extracted using Trizol reagent (Invitrogen, Waltham, MA). Library preparation, sequencing and analysis of differentially expressed RNAs were performed as described previously (2). DEGs were identified using *p* value < 0.05 and >1.5-fold change as cut-offs. Enriched pathways of DEGs were analyzed using Metascape (3).

Mouse back skin was homogenized and RNA was extracted using Quick-RNA Miniprep Kit (Zymo Research, Cat# R1054) per the manufacturer’s instructions.

For RT-qPCR, 1 μg of RNA was used to generate cDNA (Applied Biosystems, Cat# 4368814) per manufacturer’s protocol. RT-qPCR was performed using a Bio-Rad CFX96 Real-Time System and SsoAdvanced Universal SYBR® Green Supermix (Bio-Rad, Cat# 172-5271). Information for gene-specific primers used is provided in Table S17.

Public RNA-seq data of primary keratinocytes from *Ahr*^+/+^ and *Ahr*^-/-^ mice (GSE62490) (4), epidermis from normal and AD human skin (GSE120721) (5), and epidermis from newborn WT and constitutively active AhR mice (GSE72449) (6) were analyzed using the online tool GEO2R.

Human single-cell RNA-seq data were downloaded from https://zenodo.org/record/4310074#.Yd4yDdBBxPd and analyzed using Python as described previously (7).

**Western blotting**

HaCaT cells were treated with 100 μM AGX51 or DMSO for 24 hours. Protein extraction and Western blotting were performed as described previously (8). ID1 antibody (Genetex, Cat# GTX133738, 1:1000) was used.

**Single cell suspension and flow cytometry**

Single cell suspension of skin was prepared as described previously (9). In brief, to obtain epidermal cell suspension, subcutaneous fat tissue was removed from the skin and skin was treated with 0.25% trypsin with the dermis side facing down for 45 min at 37℃. Epidermis was then separated from dermis, minced using a fine scissor and filtered through a 40 μm filter. To obtain whole skin cell suspension, skin was minced using a fine scissor and then digested with 10 ml of RPMI 1640 Medium (Gibco, Cat# 11875093) containing 0.25% collagenase (Sigma, Cat# C9091), 0.01 M HEPES (Thermo Fisher Scientific, Cat# BP310), 0.001 M sodium pyruvate (Thermo Fisher Scientific, Cat# BP356), and 0.1 mg/ml DNase (Sigma, Cat# DN25) for 1-1.5 hours at 37 ℃ with rotation, and then filtered through a 40 μm filter. To obtain a single-cell suspension from skin-draining lymph nodes, the inguinal lymph nodes from mice were ground in cold PBS using the rubber tip of a syringe plunger to disrupt the tissue and release individual cells. The resulting cell suspension was then filtered through a 40 μm filter. Cells were spun down and resuspended in FACS buffer (2% fetal bovine serum in PBS). To detect intracellular IL-4 expression, cells were treated with Cell Stimulation Cocktail (plus protein transport inhibitors) (eBioscience, Cat# 00-4975-03) for 5 hours. For cell surface staining, cells were incubated with TruStain FcX™ Antibody (Biolegend, Cat# 101319) to block Fc receptors for 10 min at room temperature and incubated with antibodies for 30 min at 4 ℃. For intracellular staining, cells were incubated with Zombie NIR™ Fixable Viability Kit (Biolegend, Cat# 423105) for 30 min at 4 ℃ before cell surface staining. After cell surface staining, cells were fixed with IC fixation buffer (eBioscience, Cat# 00-8222-49) for 30 min at 4 ℃, then stained with antibodies in permeabilization buffer (eBioscience, Cat# 00-8333-56) for 30 min at 4 ℃. Cells with surface staining only were incubated with SYTOX™ Blue Dead Cell Stain (Invitrogen, Cat# S34857) for 5 min before analysis. Cells were analyzed using BD FACSAria Fusion Sorter. Data were analyzed using FlowJo v10.7.2. Antibodies used were: Brilliant Violet 510 anti-mouse CD45 (Biolegend, Cat# 103137, Clone 30-F11), allophycocyanin (APC) anti-mouse CD45 (Biolegend, Cat# 103112, Clone 30-F11), Alexa Fluor 488 anti-mouse CD11b (Biolegend, Cat# 101219, Clone M1/70), APC/Cyanine7 anti-mouse Ly6g (Tonbo Biosciences, Cat# 25-1276, Clone 1A8), APC anti-mouse CD11c (Biolegend, Cat# 117310, Clone N418), phycoerythrin (PE) anti-mouse F4/80 (Biolegend, Cat# 101219, Clone BM8), PE/Cyanine7 anti-mouse EpCAM (Biolegend, Cat# 118216, Clone G8.8), PerCP/Cyanine5.5 anti-mouse CD3 (Biolegend, Cat# 100217, Clone 17A2), Alexa Fluor 700 anti-mouse lineage (Biolegend, Cat# 133313), PE anti-mouse γδTCR (Biolegend, Cat# 118108, Clone GL3), Brilliant Violet 605 anti-mouse CD4 (Biolegend, Cat# 100548, Clone RM4-5), Fluorescein isothiocyanate (FITC) anti-mouse CD8 (Biolegend, Cat# 100706, Clone 53-6.7), PerCP/Cyanine5.5 anti-mouse CD90.2 (Biolegend, Cat# 105338, Clone 30-H12), FITC anti-mouse MHC II (Invitrogen, Cat# 11-5321-82, Clone M5/114.15.2), FITC anti-mouse IL-4 (eBioscience, Cat# 11-7042-82, Clone BVD6-24G2), and PE anti-mouse CD207 (Invitrogen, Cat# 12-2075-82, Clone eBioL31).

**SUPPLEMENTAL TABLES**

**Table S1. List of the differentially expressed genes in NHEKs treated with siNC+FICZ *versus* siNC+DMSO.**

**Table S2. Metascape result of the FICZ-induced genes.**

**Table S3. Metascape result of the FICZ-suppressed genes.**

**Table S4. List of the differentially expressed genes in NHEKs treated with si*OVOL1*+DMSO *versus* siNC+DMSO.**

**Table S5. Metascape result of the downregulated genes upon *OVOL1* depletion in DMSO-treated NHEKs.**

**Table S6. Metascape result of the upregulated genes upon *OVOL1* depletion in DMSO-treated NHEKs.**

**Table S7. List of the differentially expressed genes in NHEKs treated with si*OVOL1*+FICZ *versus* siNC+FICZ.**

**Table S8. Metascape result of the downregulated genes upon *OVOL1* depletion in FICZ-treated NHEKs.**

**Table S9. Metascape result of the upregulated genes upon *OVOL1* depletion in FICZ-treated NHEKs.**

**Table S10. List of FICZ-induced or FICZ-suppressed and OVOL1-dependent genes.**

**Table S11. Metascape result of FICZ-induced and OVOL1-dependent genes.**

**Table S12. Metascape result of FICZ-suppressed and OVOL1-dependent genes.**

**Table S13. List of the differentially expressed genes in NHEKs treated with si*AHR*+TSB *versus* siNC+TSB.**

**Table S14. List of the differentially expressed genes in NHEKs treated with si*OVOL1*+TSB *versus* siNC+TSB.**

**Table S15. List of the genes commonly regulated by AhR and OVOL1 in TSB-treated NHEKs.**

**Table S16. Metascape result of the downregulated genes upon *AHR* or *OVOL1* depletion in TSB-treated NHEKs.**

**Table S17. Metascape result of the upregulated genes upon *AHR* or *OVOL1* depletion in TSB-treated NHEKs.**

**Table S18. List of the differentially expressed genes in the epidermis of *Ovol1*^-/-^ and control mice.**

**Table S19. Metascape result of the upregulated genes in the epidermis of *Ovol1*^-/-^ mice.**

**Table S20. Metascape result of the downregulated genes in the epidermis of *Ovol1*^-/-^ mice.**

**Table S21. Annotated peaks of Ovol1 ChIP-seq analysis.**

**Table S22. Metascape result of Ovol1 target genes.**

**Table S23. List of the 125 human homologs of Ovol1 target genes that are upregulated in AD epidermis.**

**Table S24. Metascape result of the 125 human homologs of Ovol1 target genes that are upregulated in AD epidermis.**

**Table S25. Primer information.**

**SUPPLEMENTAL FIGURE LEGENDS**

**Figure S1. Metascape data of FICZ- or OVOL1-regulated genes in NHEKs. Related to Figure 1.** **(A)** Heatmap of the DEGs between NF and ND. **(B-C)** Ingenuity pathways analysis of the FICZ-induced (B) or FICZ-suppressed (C) genes. **(D)** Volcano plots showing DEGs between OD and ND. n = 2 per group. **(E)** Heatmap of the DEGs between OD and ND. **(F-G)** Ingenuity pathways analysis of the downregulated (F) or upregulated (G) genes upon *OVOL1* depletion in DMSO-treated NHEKs. **(H)** Heatmap of the DEGs between OF and NF. **(I-J)** Ingenuity pathways analysis of the downregulated (I) or upregulated (J) genes upon *OVOL1* depletion in FICZ-treated NHEKs. **(K-L)** Venn diagrams of DEGs showing overlap between *AHR* depletion and *OVOL1* depletion. Down, downregulated. UP, upregulated. Numbers of DEGs are as indicated. **(M-N)** Ingenuity pathways analysis of the common downregulated (M) or upregulated (N) genes upon *OVOL1* depletion or *AHR* depletion in TSB-treated NHEKs. ND: siNC + DMSO, NF: siNC + FICZ, OD: si*OVOL1* + DMSO, OF: si*OVOL1* + FICZ.

**Figure S2. Impact of *Ahr* or *Ovol1* deletion on the expression of previously reported AhR targets. Related to Figure 2. (A)** RNA-seq results for the indicated genes in primary keratinocytes from *Ahr*^+/+^ (WT, n = 4) or *Ahr*^-/-^ (KO, n = 3) mice. **(B)** Venn diagram showing the overlapping upregulated genes between the AhR-CA epidermis and the siNC + FICZ (NF)-treated NHEKs. **(C)** RNA-seq results (GSE120721) of *AHR* expression in the epidermis of healthy (NC, n = 10) and AD lesional (n = 5) human skin. **(D)** Immunostaining of AhR protein in healthy and AD lesional skin. n = 10 samples per group. Scale bar = 50 μm. **(E)** Immunostaining of AhR protein in the back skin of DMSO-treated *Ahr* WT and *Ahr* cKO mice at day 11. n = 3 samples per group. Scale bar = 50 μm. **(F)** Time course showing TEWL measurements. n = 3-5 mice per group. **(G)** Representative skin histology (H/E staining) at day 11. Scale bar = 100 μm. **(H)** Quantification of epidermal thickness. n = 3-5 mice per group. **(I)** Relative expression of *Cyp1a1* mRNA in DMSO- or FICZ-treated Ctrl mice. The expression level in DMSO-treated mice was normalized to 1. Data are mean + SEM. n = 4 mice per group. **(J)** RT-qPCR results of the indicated genes in the whole skin of control (Ctrl) and SSKO mice at day 11 after FICZ or DMSO treatment. Data are mean + SEM. n = 4 mice per group. **(K)** Genome browser track for the indicated ChIP-seq signals across the *Cyp1b1* locus. Green box highlights the Ovol1-bound region. **(L)** ChIP-qPCR results of *Cyp1b1* in mouse keratinocytes treated with Ca^2+^ (1.8 mM) for 24 hours. IgG control values were normalized to 1. Data are mean + SEM. Results are summarized from 3 independent experiments. **(M)** Working model of the regulation of *Cyp1b1* by AhR and Ovol1. DAPI in (D) and (E) stains the nuclei. * *p* < 0.05, ** *p* < 0.01, *** *p* < 0.001. *p* values were calculated using 2-tailed unpaired Student *t* test (A, C, I and L) or two-way ANOVA (F, H and J).

**Figure S3. *Ovol1* deficiency in keratinocytes aggravates AD-like skin inflammation in mice. Related to Figure 3. (A)** Feature plots showing expression of the indicated genes in all cells of healthy and AD lesional and non-lesional skin. **(B)** Clinical score of skin eruption, scaling, bleeding and redness in HDM/SEB-treated Ctrl (n = 8) and SSKO (n = 7) mice at day 11. **(C)** Eosinophil counts in the epidermis of HDM/SEB-treated Ctrl and SSKO mice at day 11. Each value represents the average of 3 to 5 high-power fields (HPF). **(D-E)** The percent of Th2 cells per CD4^+^ T cells (D) in skin-draining lymph nodes (LN) and the total number of Th2 cells per LN (E). n = 3 mice per group. **(F)** Representative immunofluorescence images of CD3 and GATA3 in lesional skin of HDM/SEB-treated Ctrl and SSKO mice at day 11. n = 4 mice per group. Scale bar = 50 μm. **(G)** Representative photographs of HDM/SEB-treated Ctrl and SSKO mice at day 31. **(H)** TEWL values of HDM/SEB-treated Ctrl and SSKO mice at day 31. n = 6 mice per group. **(I)** Clinical scores of HDM/SEB-treated Ctrl and SSKO mice at day 31. n = 6 mice per group. **(J)** Representative skin histology (H/E staining) HDM/SEB-treated Ctrl and SSKO mice at day 31. Scale bar = 100 μm. **(K)** Quantification of epidermal, dermal and total skin thickness. n = 3 mice per group. **(L)** Representative skin histology (H/E staining) of untreated Ctrl and SSKO mice. Scale bar = 100 μm. **(M)** Volcano plots showing differential gene expression in the epidermis of untreated *Ovol1*^-/-^ and control mice. **(N-O)** Ingenuity pathways analysis of the upregulated (D) or downregulated (E) genes in *Ovol1*^-/-^ epidermis. **(P)** Mean ± SEM ear thickness of MC903-treated control (Ctrl) and *Ovol1*^-/-^ mice. n = 6 mice per group. **(Q)** Representative skin histology (H/E staining) of MC903-treated Ctrl and *Ovol1*^-/-^ mice at day 15. Scale bar = 100 μm. **(R)** Thickness of sham (ethanol-treated) ear skin at 9 days after MC903 application to the other ear of the same mouse (n = 6 per group) or in untreated (n = 3 per group) mice. **(S)** Ki67 and K14 immunostaining of sham-treated, left ears of mice that were treated with MC903 on their right ears. Scale bar = 50 µm. **(T)** Thickness of back skin epidermis in mice that were treated with MC903 on their right ears. n = 3 mice per group. **(U)** Serum ELISA analysis of mice that were treated with MC903 on their right ears. n = 3 mice per group. **(V-W)** Ly6G and K14 immunostaining of the MC903-treated right ears (V) and ethanol-treated (W) left ears. White arrows point to neutrophils that are present on the skin surface (V) or in dermis (W). Scale bar = 50 µm. DAPI in (F), (V) and (W) stains the nuclei. * *p* < 0.05, ** *p* < 0.01, *** *p* < 0.001, **** *p* < 0.0001. *p* values were calculated using two-way ANOVA (P) or 2-tailed unpaired Student *t* test (B, C, D, E, H, I, K, R, T and U).

**Figure S4. Supporting evidence for Ovol1 target analysis in epidermal keratinocytes. Related to Figure 4. (A)** Venn diagram of Ovol1 target genes and genes upregulated in the OD (si*OVOL1* + DMSO)-treated NHEKs compared to ND (siNC + DMSO)-treated NHEKs. **(B)** Venn diagram of Ovol1 target genes and genes upregulated in the IMQ-treated *Ovol1* null epidermis compared to control littermate. For both (A) and (B), gene numbers are indicated in the Venn diagram, and overlapping genes are listed on the left. **(C)** Ingenuity pathways analysis of the 39 overlapping genes from (B). **(D)** RNA-seq results for the indicated genes in tryptic soy broth (TSB)-treated NHEKs with or without *OVOL1* depletion. **(E)** RT-qPCR results of the indicated genes in DMSO- or FICZ-treated NHEKs with or without OVOL1 knockdown. Results are summarized from 3 independent experiments. * *p* < 0.05, *** *p* < 0.001. *p* values were calculated using 2-tailed unpaired Student *t* test (D) or two-way ANOVA (E). OD: si*OVOL1* + DMSO, ND: siNC + DMSO.

**Figure S5. Supporting evidence for *Id1* as a functional target of Ovol1 in AD-like skin inflammation. Related to Figure 5.** **(A)** Western blotting results of ID1 expression in HaCaT cells treated with DMSO or AGX51 (100 μM) for 24 hours. Results are summarized from 3 independent experiments. **(B)** Clinical scores of skin eruption, scaling, bleeding and redness of DMSO- or AGX51-treated SSKO mice at day 11. **(C)** Quantification of dermal and total skin thickness. n = 5-6 mice per group for (B-C). **(D)** TEWL values of DMSO- or AGX51-treated WT mice at day 11. n = 5 mice per group. **(E)** Representative skin histology (H/E staining) of DMSO- or AGX51-treated WT mice at day 11. Scale bar = 100 μm. **(F)** Quantification of epidermal thickness in DMSO- or AGX51-treated WT mice. n = 5 mice per group. * *p* < 0.05. *p* values were calculated using 2-tailed unpaired Student *t* test.

**Figure S6. Immune cell profiles of Ctrl and SSKO mice under homeostatic condition. Related to Figure 5. (A)** Representative flow cytometry plots showing gating strategy for LCs (1) and T cells (2) within the epidermis. **(B)** Percentages of CD45^+^ cells, LCs, and T cells out of live epidermal cells. **(C)** Percentages of LCs and T cells out of epidermal CD45^+^ cells. For (B-C), n = 11 mice for Ctrl group; n = 6 mice for SSKO group. **(D)** Representative flow cytometry plots showing gating strategy for T cells (3), CD4^+^ T cells (4), CD8^+^ T cells (5) and CD4^-^CD8^-^ (γδ) T cells (6) within the dermis. **(E)** Percentages of the indicated cell types out of live epidermal cells. **(F)** Percentages of the indicated cell types dermal CD45^+^ cells. For (E-F), n = 5 mice for Ctrl group; n = 3 mice for SSKO group. *p* values were calculated using 2-tailed unpaired Student *t* test.

**Figure S7. Immune cell profiles of the HDM/SEB-treated Ctrl and SSKO mice and the effect of Id1 inhibition on immune cell infiltration. Related to Figure 5. (A)** Representative flow cytometry plots showing gating strategy for (1) T cells, (2) neutrophils, (3) LCs, (4) macrophages, (5) DCs, and (6) other CD11b^+^ cells. **(B)** Representative flow cytometry plots showing gating strategy for (7) dermal γδT cells, (8) DETCs, (9) ILCs, (10) CD4^+^ T cells, and (11) CD8^+^ T cells. **(C)** Percentages of different immune cell populations out of total skin cells from mice treated with HDM/SEB at day 11. **(D)** Percentages of different immune cell populations out of skin CD45^+^ cells from mice treated with HDM/SEB at day 11. n = 5-6 mice per group (F, G). **(E-F)** tSNE visualization of different immune cell populations in mice treated with HDM/SEB at day 31. **(G)** Quantification of the indicated immune cell types per gram of skin tissue from mice treated with HDM/SEB at day 31. n = 3 mice per group. **(H-I)** Percentages of different immune cells out of total skin cells (H) or skin CD45^+^ cells (I) from mice treated with HDM/SEB at day 31. n = 3 mice per group. **(J)** Quantification of the indicated immune cell types per gram of skin tissue in DMSO- or AGX51-treated SSKO mice at day 11. n = 4 mice per group. **(K-L)** Percentages of different immune cell populations out of total skin cells (K) or skin CD45^+^ cells (L) at day 11. n = 4-5 mice per group. **(M)** Quantification of the indicated immune cell types per gram of skin tissue in DMSO- or AGX51-treated WT mice at day 11. n = 4 mice per group. * *p* < 0.05, ** *p* < 0.01. *p* values were calculated using 2-tailed unpaired Student *t* test.

**Figure S8. Supporting evidence for the importance of γδT cells in skin inflammation of HDM/SEB-treated SSKO mice. Related to Figure 6. (A)** Clinical scores of skin eruption, scaling, bleeding and redness in IgG- or γδTCR Ab-treated SSKO mice at day 11. **(B)** Quantification of dermal and total skin thickness in IgG- or γδTCR Ab-treated SSKO mice at day 11. **(C)** Representative skin histology (H/E staining) of IgG- or γδTCR Ab-treated WT mice at day 11. Scale bar = 100 μm. **(D)** Quantification of epidermal thickness of IgG- or γδTCR Ab-treated WT mice. n = 4 mice per group. **(E)** TEWL values of IgG- or γδTCR Ab-treated WT mice at day 11. n = 4 mice per group. **(F)** Quantification of the indicated immune cell populations per gram of skin tissue of IgG- or γδTCR Ab-treated SSKO mice at day 11. n = 5 mice per group. **(G-H)** Percentages of different immune cells out of total skin cells (G) or skin CD45^+^ cells (H) in IgG- or γδTCR Ab-treated SSKO mice at day 11. n = 5 mice per group. * *p* < 0.05, ** *p* < 0.01, *** *p* < 0.001. *p* values were calculated using 2-tailed unpaired Student *t* test.

**Figure S9. Supporting evidence for the importance of IL-1 signaling in skin inflammation of HDM/SEB-treated SSKO mice. Related to Figure 6. (A-D)** Flow cytometry analysis of the indicated cell types in the back skin of IgG- or IL-1R Ab-treated SSKO mice. See Fig. 5 and S7 legends for additional details. n = 4 mice per group. **(E)** Quantification of the indicated immune cell populations per gram of skin tissue in IgG- or IL-1R Ab-treated SSKO mice at day 11. See Fig. 5 and S7 legends for additional details. n = 4 mice per group. **(F)** Clinical scores of skin eruption, scaling, bleeding and redness in IgG- or IL-1R Ab-treated SSKO mice at day 11. n = 5 mice per group. **(G)** Flow cytometry analysis of dermal γδT cells in the back skin of IgG- or IL-1R Ab-treated WT mice. See Fig. 5 legends for additional details. n = 5 mice per group. **(H)** Representative skin histology (H/E staining) of IgG- or IL-1R Ab-treated WT mice at day 11. Scale bar = 100 μm. **(I)** Quantification of epidermal thickness in IgG- or IL-1R Ab-treated WT mice. n = 5 mice per group. **(J)** Quantification of dermal and total skin thickness in IgG- or IL-1R Ab-treated SSKO mice at day 11. n = 4 mice per group. **(K)** TEWL values in IgG- or γδTCR Ab-treated WT mice at day 11. n = 4 mice per group. **(L)** RT-qPCR analysis of the indicated genes in whole skin of IgG- or IL-1R Ab-treated SSKO mice at day 11. n = 5 mice per group. * *p* < 0.05, ** *p* < 0.01, *** *p* < 0.001. *p* values were calculated using 2-tailed unpaired Student *t* test.

**Figure S10. Supporting evidence for the importance of AhR/OVOL1/ID1 axis in AD and psoriasis (PS). Related to Figure 7. (A)** Genome browser tracks show indicated ChIP-seq signals across the indicated Ovol1 target gene loci. Red boxes highlight the Ovol1-bound regions. **(B)** Immunostaining of AhR protein in healthy, PS lesional and AD lesional skin. Scale bar = 50 μm. **(C)** Expression of *OVOL1* RNA in healthy, PS lesional and AD lesional skin. Scale bar = 50 μm. **(D-E)** Immunostaining of ID1 protein (D) and AQP3 protein (E) in healthy, PS lesional and AD lesional skin. Scale bar = 50 μm. For (B-E), n = 10 for healthy group; n = 5 for PS group; n = 10 for AD group. Dashed lines in (B) and (D) indicate the basement membrane, and DAPI in (B-E) stains the nuclei.

**REFERENCES**

1. Folmsbee M, Howard G, McAlister M. Nutritional effects of culture media on mycoplasma cell size and removal by filtration. Biologicals. 2010;38(2):214-7.

2. Yu Z, Gong Y, Cui L, Hu Y, Zhou Q, Chen Z, et al. High-throughput transcriptome and pathogenesis analysis of clinical psoriasis. Journal of dermatological science. 2020;98(2):109-18.

3. Zhou Y, Zhou B, Pache L, Chang M, Khodabakhshi AH, Tanaseichuk O, et al. Metascape provides a biologist-oriented resource for the analysis of systems-level datasets. Nat Commun. 2019;10(1):1523.

4. van den Bogaard EH, Podolsky MA, Smits JP, Cui X, John C, Gowda K, et al. Genetic and pharmacological analysis identifies a physiological role for the AHR in epidermal differentiation. J Invest Dermatol. 2015;135(5):1320-8.

5. Esaki H, Ewald DA, Ungar B, Rozenblit M, Zheng X, Xu H, et al. Identification of novel immune and barrier genes in atopic dermatitis by means of laser capture microdissection. J Allergy Clin Immunol. 2015;135(1):153-63.

6. Hidaka T, Ogawa E, Kobayashi EH, Suzuki T, Funayama R, Nagashima T, et al. The aryl hydrocarbon receptor AhR links atopic dermatitis and air pollution via induction of the neurotrophic factor artemin. Nat Immunol. 2017;18(1):64-73.

7. Reynolds G, Vegh P, Fletcher J, Poyner EFM, Stephenson E, Goh I, et al. Developmental cell programs are co-opted in inflammatory skin disease. Science. 2021;371(6527).

8. Yu Z, Yu Q, Xu H, Dai X, Yu Y, Cui L, et al. IL-17A Promotes Psoriasis-Associated Keratinocyte Proliferation through ACT1-Dependent Activation of YAP-AREG Axis. J Invest Dermatol. 2022;142(9):2343-52.

9. Dragan M, Sun P, Chen Z, Ma X, Vu R, Shi Y, et al. Epidermis-Intrinsic Transcription Factor Ovol1 Coordinately Regulates Barrier Maintenance and Neutrophil Accumulation in Psoriasis-Like Inflammation. J Invest Dermatol. 2022;142(3 Pt A):583-93 e5.
