# Supplementary figures and images for: The AhR-Ovol1-Id1 regulatory axis in keratinocytes promotes epidermal and immune homeostasis in atopic dermatitis-like skin inflammation
Source: Cell Mol Immunol. 2025 Feb 13;22(3):300–15. doi: 10.1038/s41423-025-01264-z (PMC11868582; doi:10.1038/s41423-025-01264-z)

Figure S1

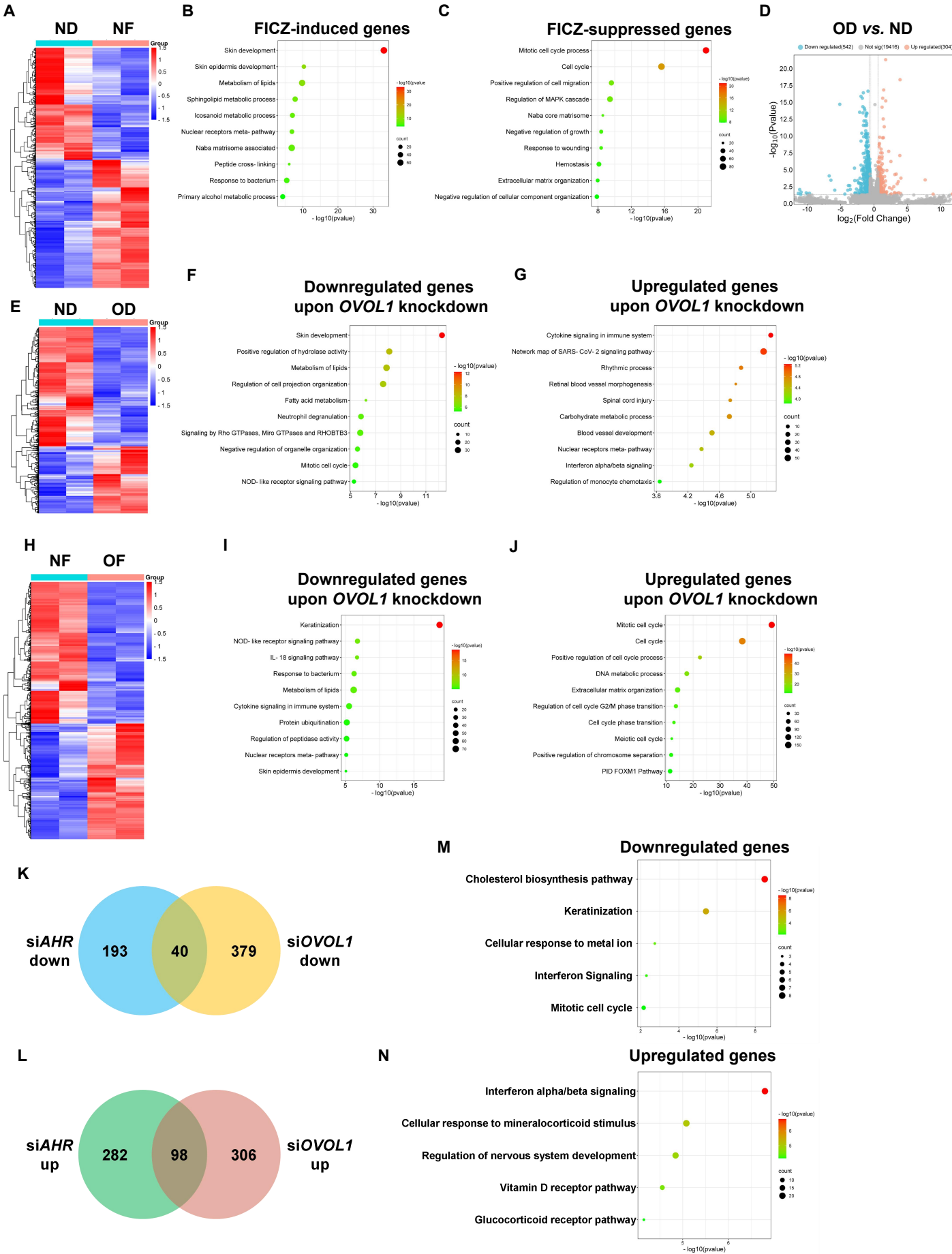

**Figure S2**

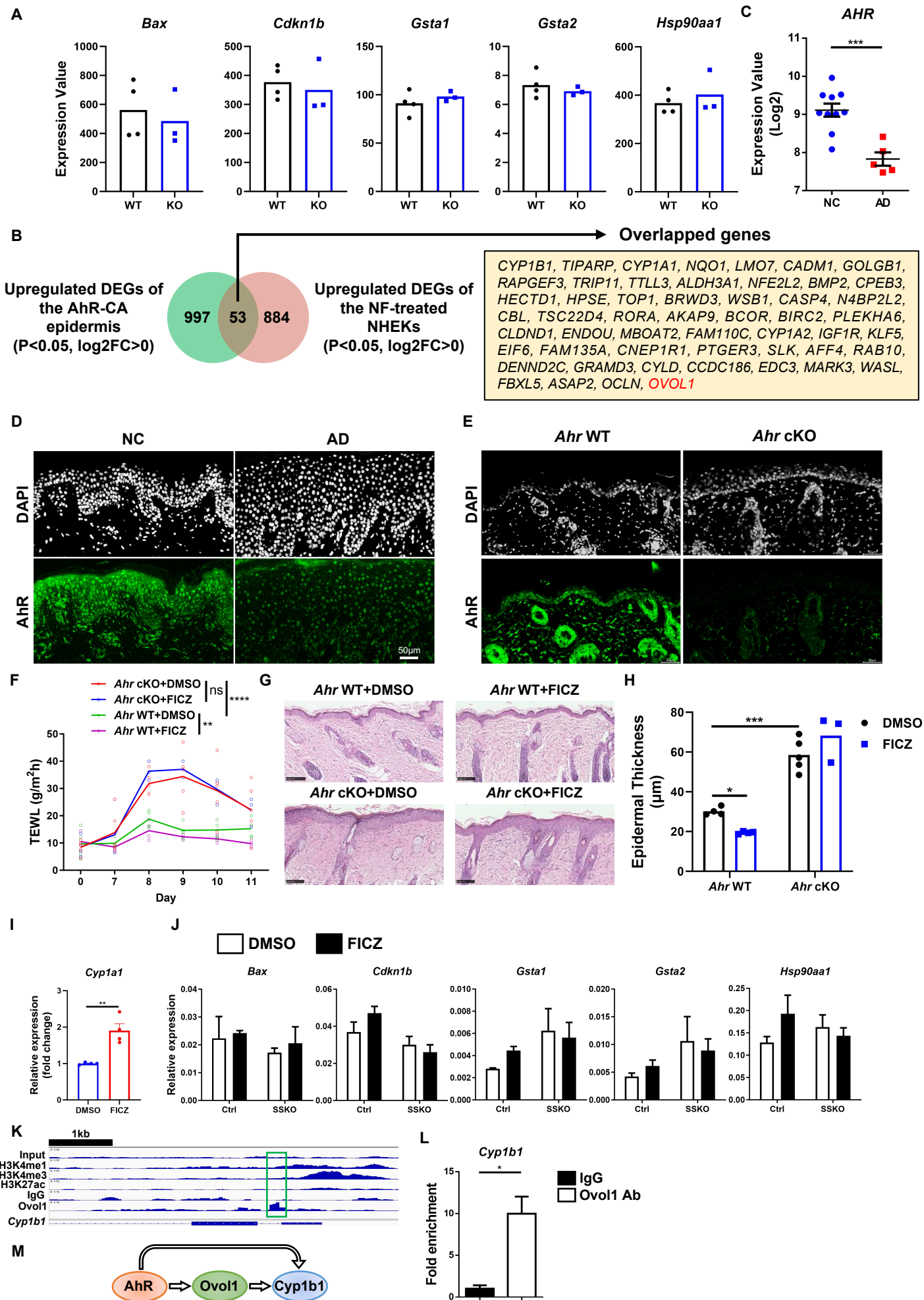

**Figure S3**

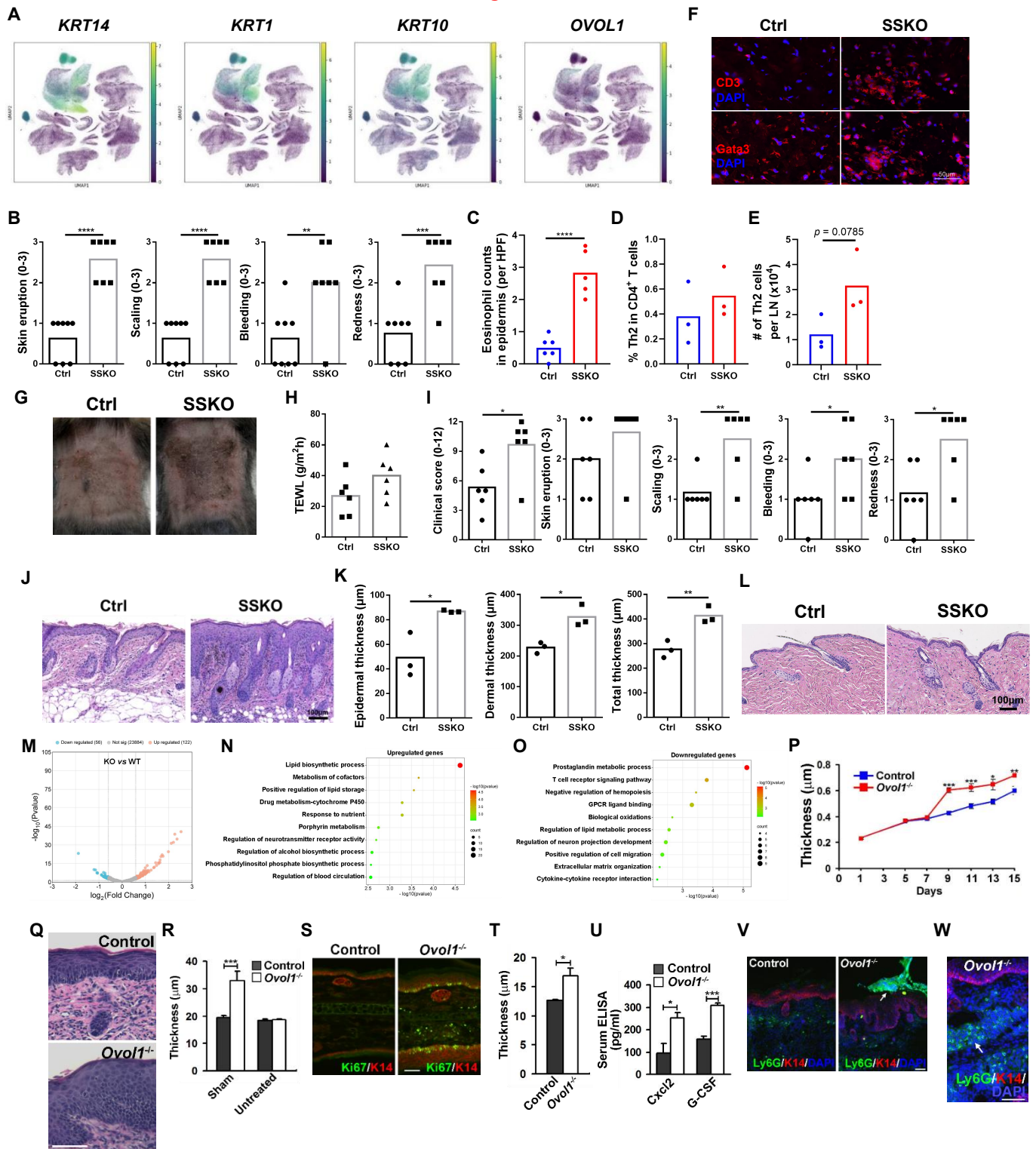

Figure S4

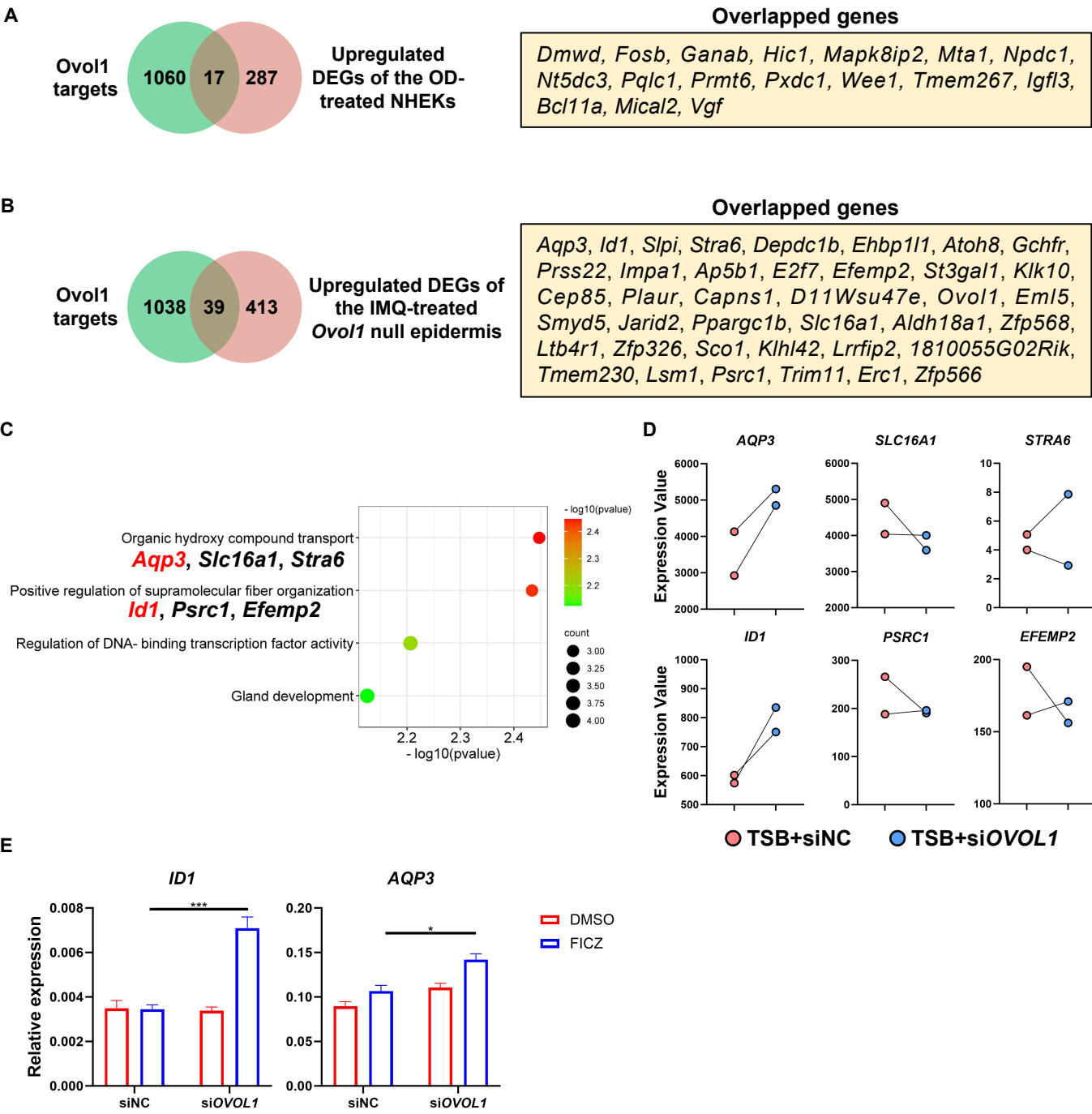

Figure S5

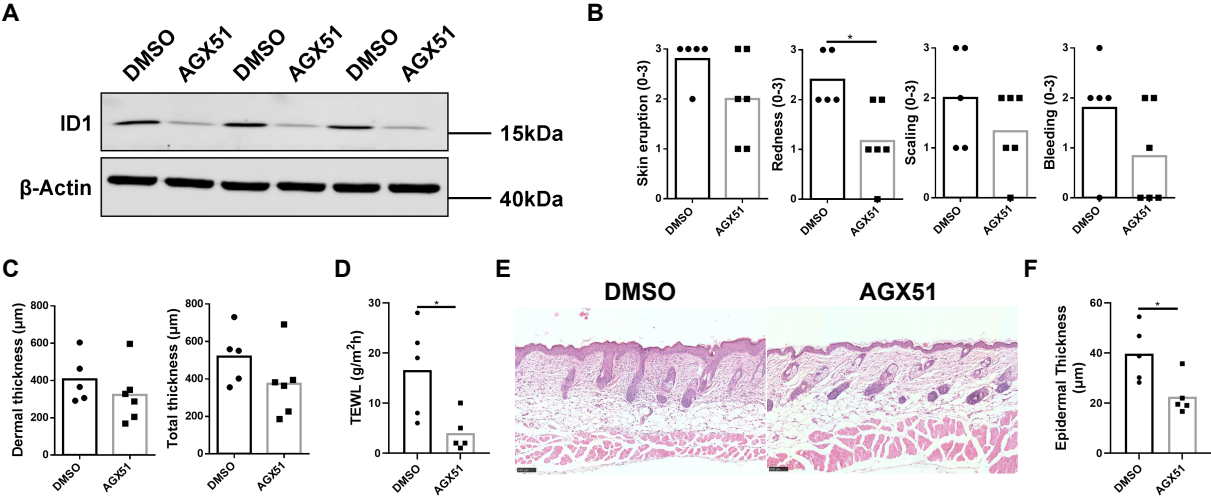

Figure S6

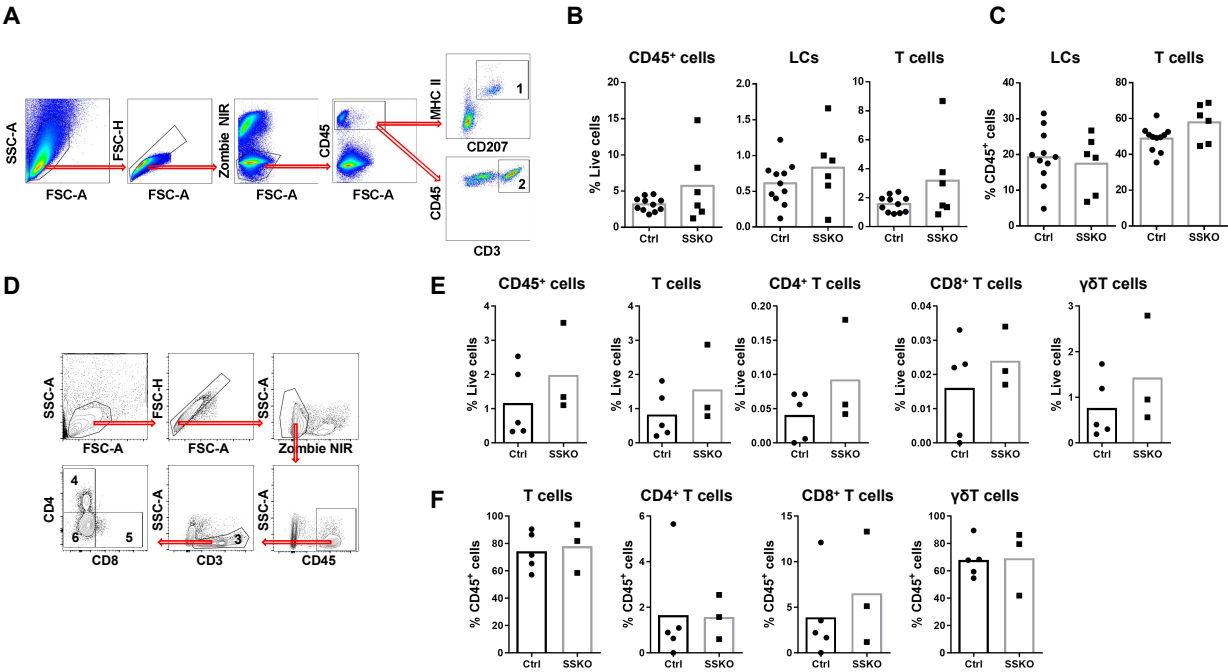

**Figure S7**

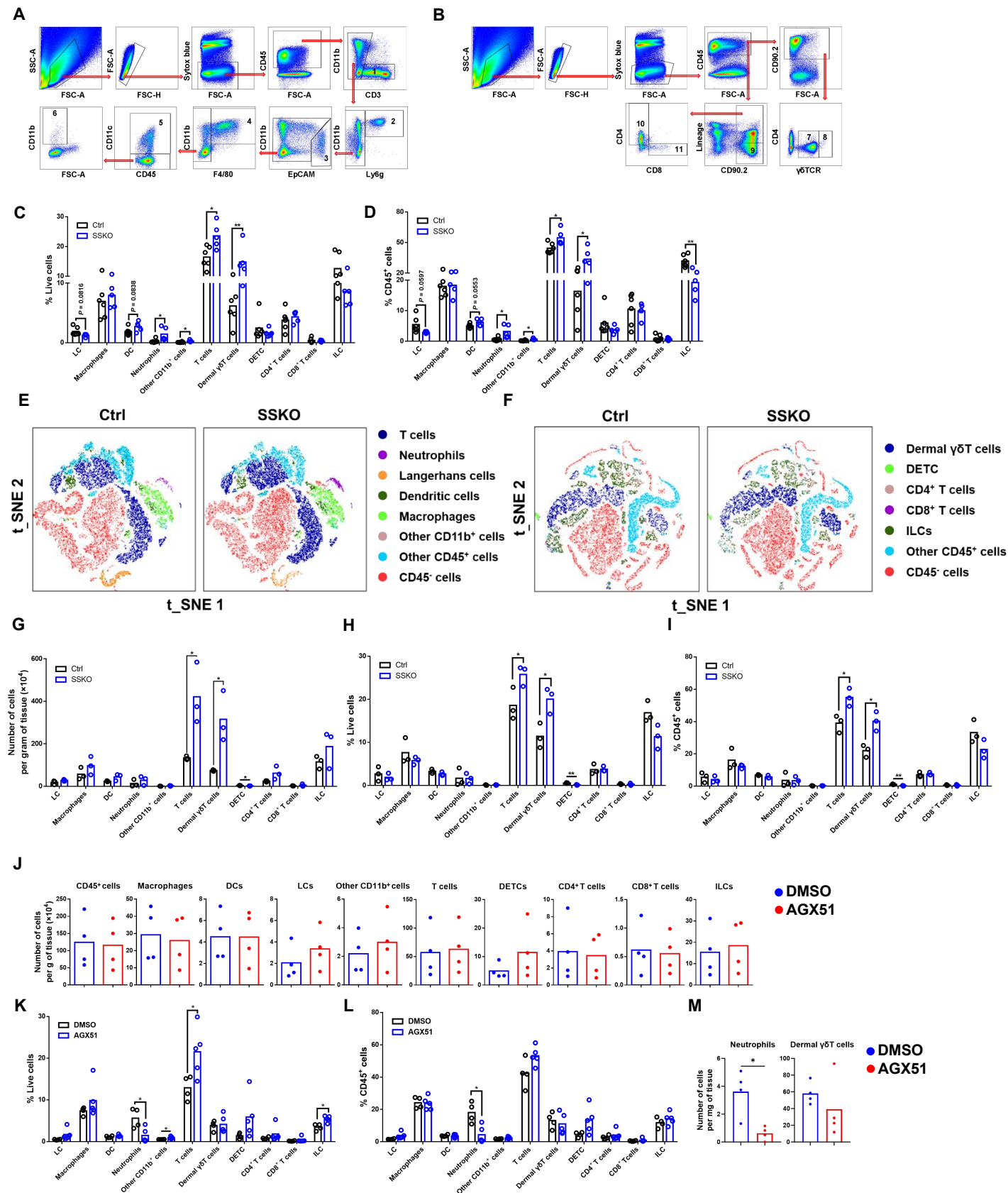

**Figure S8**

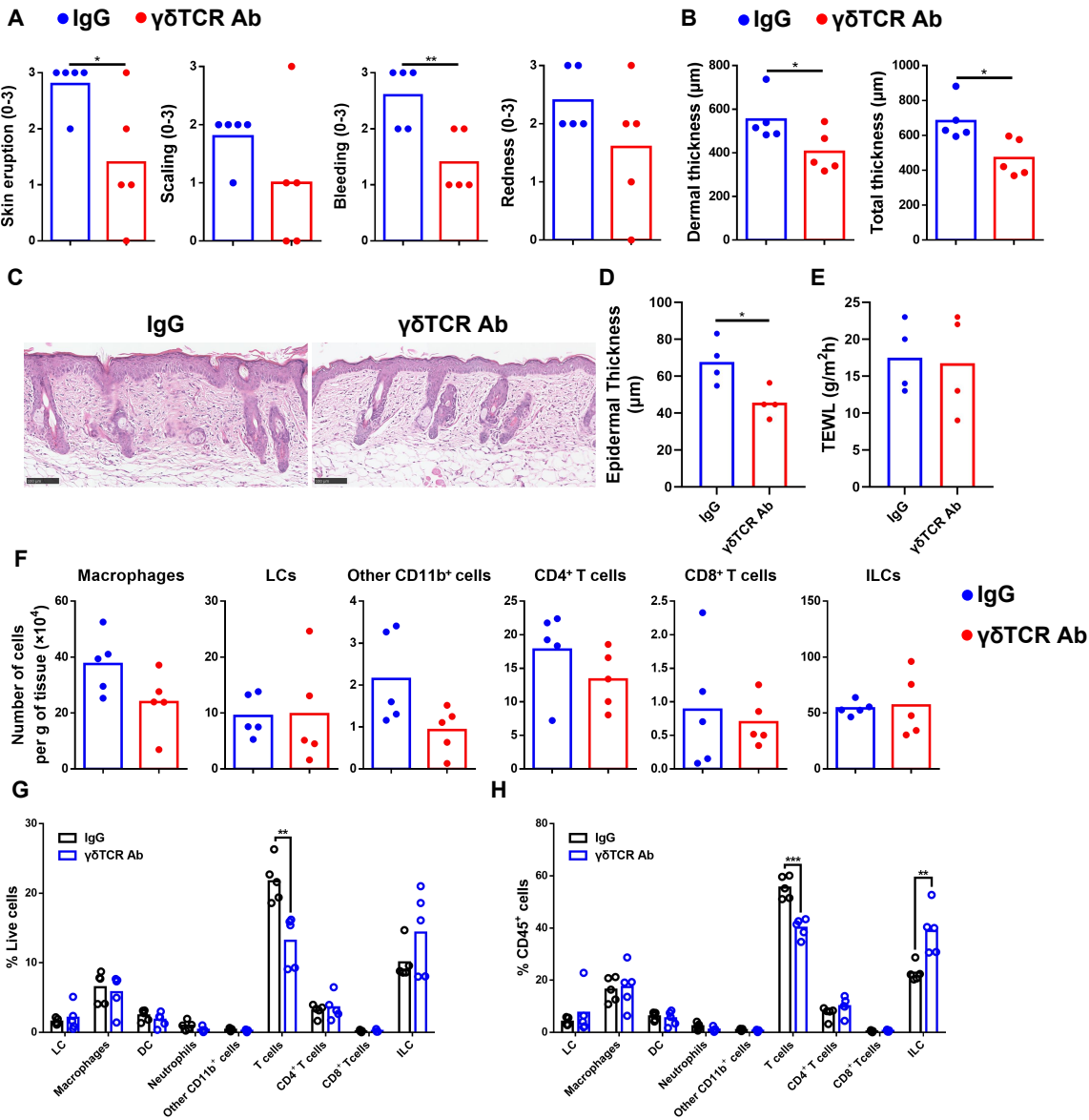

**Figure S9**

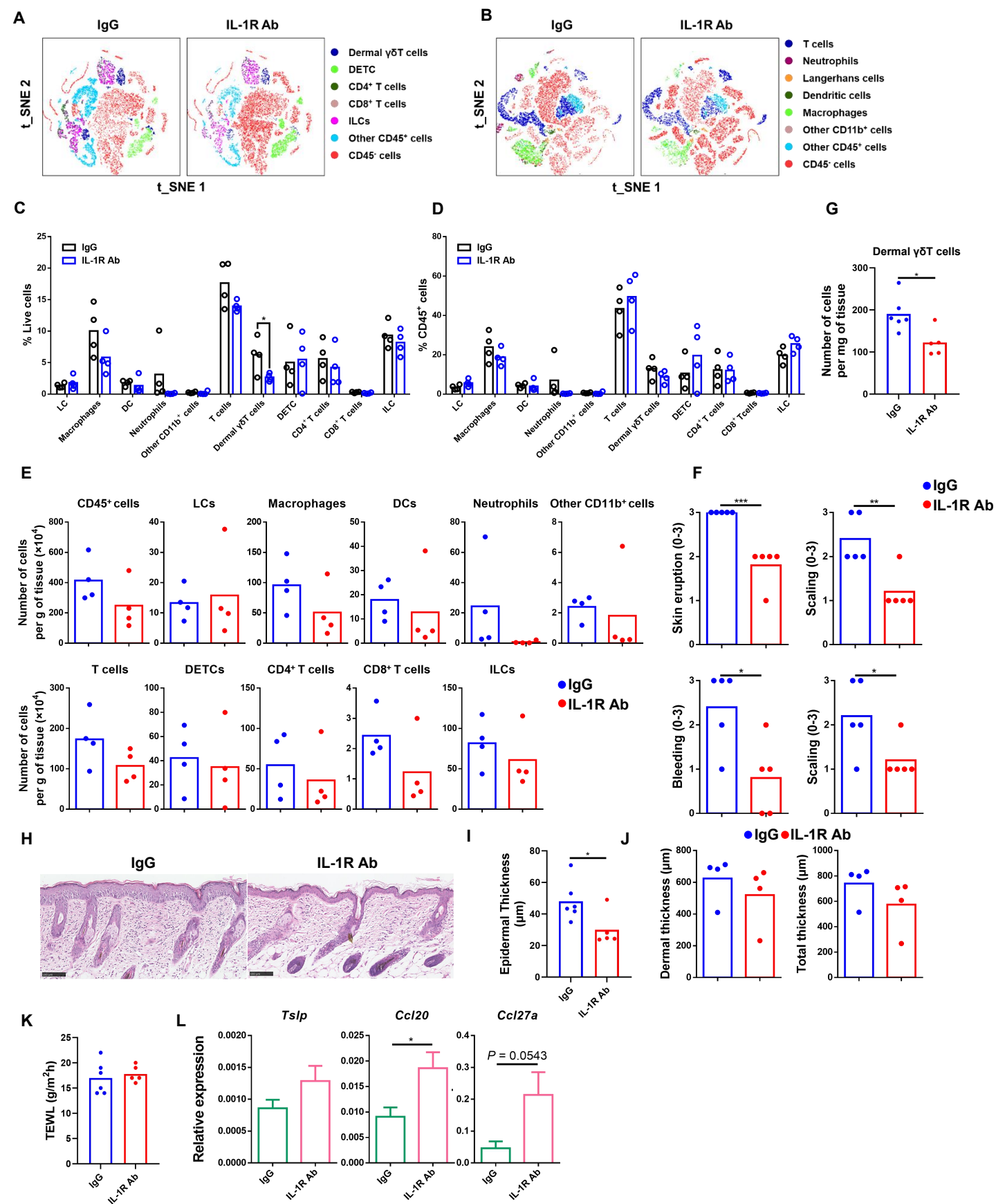

Figure S10

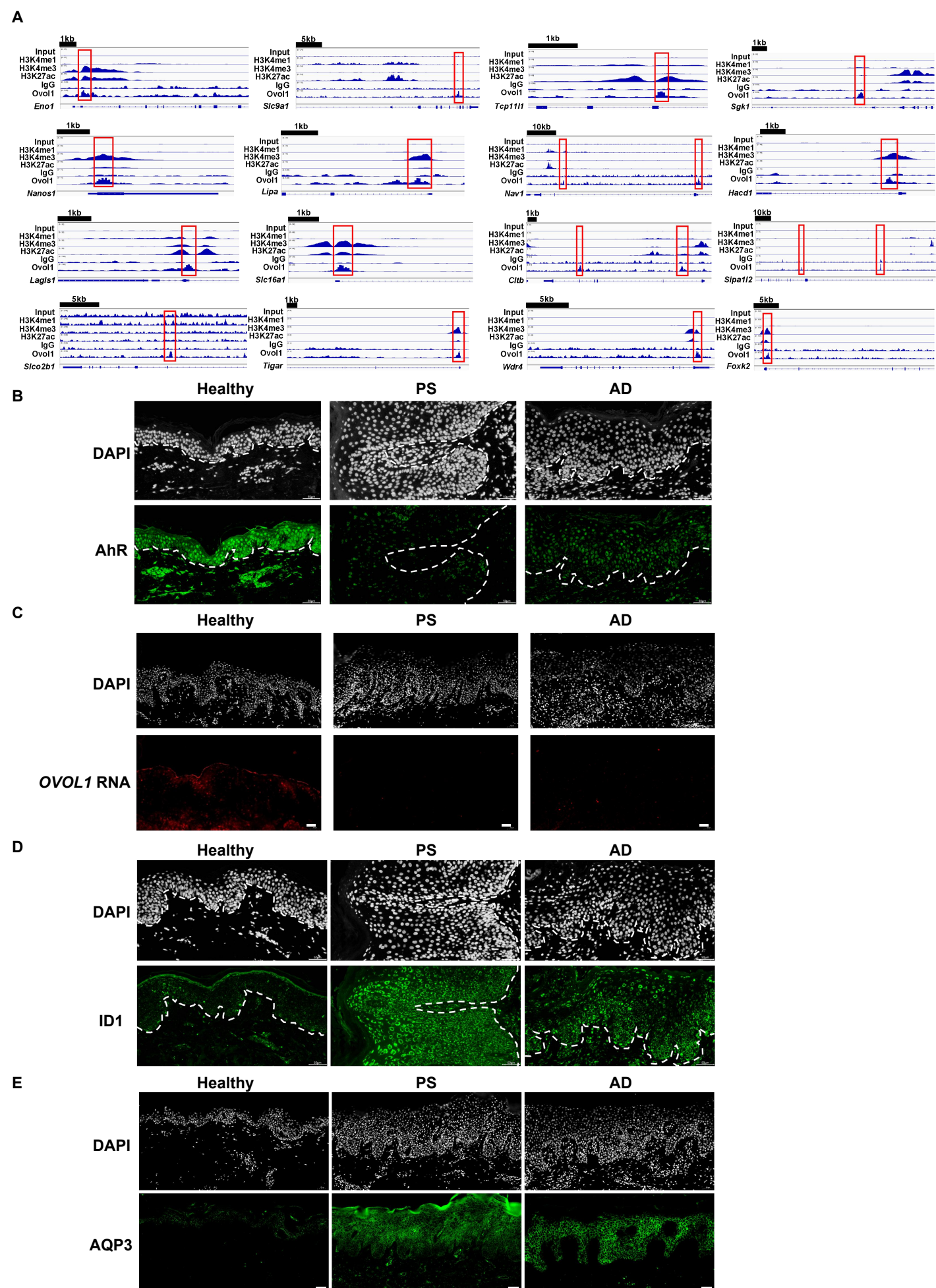

Supplement: Supplementary file 2 — Supplemental Figures [file 41423_2025_1264_MOESM2_ESM.pdf]

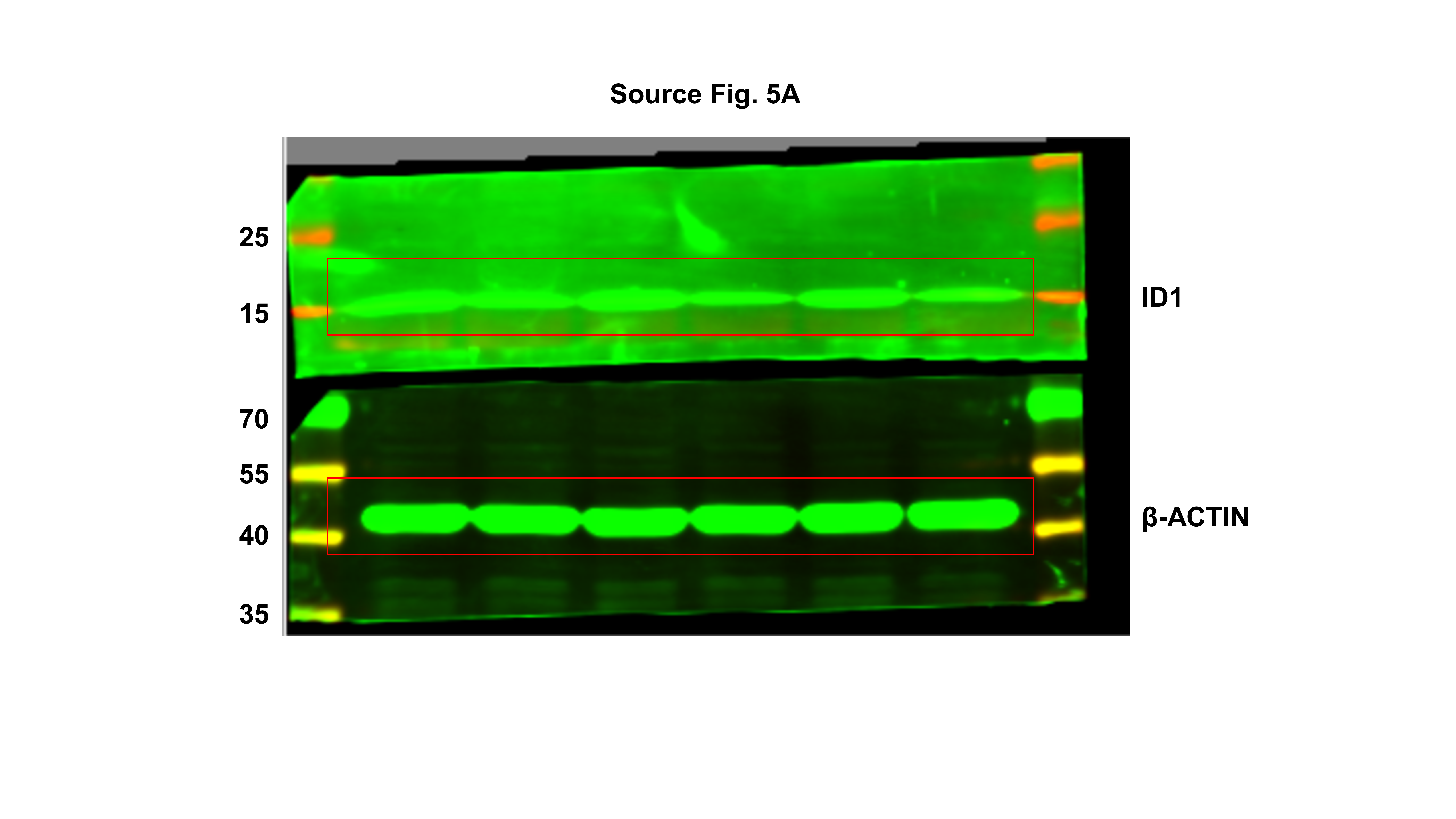

Supplement: Supplementary file 3 — Source Figure S5A [file 41423_2025_1264_MOESM3_ESM.tif]
